# From Gene Copies to Cell Numbers: Advancing Quantitative Approaches in Protistan Ecology Using Digital PCR

**DOI:** 10.1111/1755-0998.70177

**Published:** 2026-07-02

**Authors:** Megan Gross, Ulrike Koll, Bettina Sonntag, Thorsten Stoeck

**Affiliations:** ^1^ Department of Ecology Rheinland‐Pfälzische Technische Universität Kaiserslautern‐Landau Kaiserslautern Germany; ^2^ Research Department for Limnology University of Innsbruck Mondsee Austria

**Keywords:** CARD‐FISH, dPCR, environmental DNA, protist, quantification, rRNA

## Abstract

Quantifying abundances of unicellular eukaryotes (protists) remains a central challenge in microbial ecology, as methodological differences can strongly influence abundance estimates and ecological interpretation. Although molecular tools have thus far greatly improved our understanding of protists, high rRNA gene copy numbers limit quantitative inferences. Digital PCR (dPCR) has emerged as a promising tool for absolute quantification, yet its application for unicellular eukaryotes and its comparability to established cell‐based methods remain insufficiently explored. Here, we develop species‐specific dPCR assays for two important freshwater ciliates (*Urotricha castalia* and *Urotricha pseudofurcata*) and establish gene copy number correction factors to enable highly accurate quantitative abundance estimates. We assess assay performance using controlled laboratory experiments and apply the approach to environmental samples, directly benchmarking dPCR against catalyzed reporter deposition‐FISH (CARD‐FISH). Under controlled conditions, dPCR and CARD‐FISH yielded comparable accuracy, with dPCR showing superior precision. In field applications, method‐dependent differences emerged, reflecting both methodological constraints and biological variability. Notably, dPCR provided an overall higher sensitivity, enabling robust detection of low‐abundance taxa. Our results highlight dPCR as a scalable and sensitive approach that, when combined with appropriate correction strategies, represents a significant step towards more reliable molecular quantification of protists. At the same time, differences between methods underscore the value of integrating molecular and microscopy‐based approaches. We propose that combining dPCR with tools such as CARD‐FISH can offer complementary insights into protist population dynamics. Such integrative frameworks provide a powerful path forward for improving abundance estimates and advancing quantitative microbial ecology.

## 1 Introduction

Protists play fundamental roles in aquatic ecosystems, mediating energy transfer, nutrient cycling, and thereby driving microbial population dynamics (Mitra et al. 2014; Singer et al. 2021; Weisse et al. 2016; Worden et al. 2015). Current estimations suggest that only a small fraction of this unicellular eukaryotic diversity and their ecological roles in the environment have been characterized (Bjorbækmo et al. 2020; Jamy et al. 2025; Santoferrara et al. 2020). While substantial progress over the past decades has helped unravel this ‘black box’ of protist diversity and led to the discovery of numerous previously undescribed phylogenetic lineages, comparable efforts to improve quantitative approaches have remained limited. Yet, understanding the role of protists in ecosystem functioning is inherently linked to resolving their spatial and temporal distributions in the environment (Frantal et al. 2022; Weisse and Montagnes 2022). Although a variety of approaches are available, accurately quantifying protists in natural communities remains challenging (Santoferrara et al. 2020; Stern et al. 2018; Weisse and Montagnes 2022). Traditional microscopy‐based methods often require extensive taxonomic expertise and are difficult to apply at high throughput, while many molecular approaches, including high‐throughput sequencing (HTS), primarily provide relative rather than absolute abundance estimates (Boscaro et al. 2017; Pitsch et al. 2019; Santoferrara et al. 2016; Stoeck et al. 2014). Consequently, translating detection signals or molecular marker data into accurate estimates of organismal abundance in high‐throughput studies remains a central methodological challenge in microbial ecology, particularly for protists with complex life cycles and variable gene copy numbers.

In recent years, fluorescence in situ hybridization techniques (FISH), particularly catalyzed reporter deposition FISH (CARD‐FISH), have emerged as valuable tools for detecting and enumerating specific microbial taxa by targeting ribosomal RNA within intact cells, effectively combining microscopy with molecular techniques (Dirren‐Pitsch et al. 2022; Piwosz et al. 2021). In CARD‐FISH, oligonucleotide probes labelled with horseradish peroxidase hybridize to complementary rRNA sequences within ribosomes and fluorescently labelled tyramides are enzymatically deposited at the probe site, amplifying the signal and enabling visualization of target cells. This enables a highly specific visualization and has already successfully been applied for enumerating various protist target taxa (Dirren‐Pitsch et al. 2022; Mukherjee et al. 2017; Pernice et al. 2015). However, the workflow remains labour‐intensive, can be sensitive to sample fixation and probe performance, and typically only small sample volumes can be processed, which may hamper the detection of rare or low‐abundant taxa (Dirren‐Pitsch et al. 2022; Piwosz et al. 2021).

To circumvent these limitations, many studies have employed HTS, which enables parallel processing of numerous samples with larger effective sampling volumes in considerably less time. This approach has not only revealed an unprecedented diversity of protists and greatly advanced our understanding of community composition and spatio‐temporal dynamics (Burki et al. 2021; De Vargas et al. 2015; Massana et al. 2015) but has also facilitated the detection of cryptic or rare taxa (Dunthorn et al. 2014). However, determining reliable abundance estimates from HTS data remains challenging because quantification is typically inferred from read numbers of marker genes, most commonly the 18S SSU rRNA gene, which occurs in multiple copies within a single genome and varies extensively both between and within species (Fu and Gong 2017; Galluzzi et al. 2010; Godhe et al. 2008; Gross et al. 2024; Wang et al. 2020). This variation complicates the translation of sequencing reads into reliable estimates of organismal abundance (Frantal et al. 2022; Lavrinienko et al. 2021; Medinger et al. 2010; Pröschold et al. 2021; Spanner et al. 2022; Sonntag et al. 2011; Stoeck et al. 2014). Consequently, developing robust methods that allow accurate and scalable quantification of protists remains a central challenge in microbial ecology.

Digital PCR (dPCR) has the potential to overcome the shortcomings of both methods by providing a highly sensitive and scalable approach capable of both high‐throughput processing of environmental samples and absolute quantification of gene copy numbers, which can theoretically be used to infer abundances of even rare taxa across a range of environmental sample volumes. While sharing similarities with quantitative PCR (qPCR), dPCR does not rely on measurements from a single bulk reaction (Wood et al. 2019). Instead, the reaction mixture is partitioned into thousands of individual subreactions, allowing gene copy numbers within a sample to be inferred from the proportion of positive partitions. By distributing the template DNA across many partitions, target molecules are physically separated and inhibitors are effectively diluted, reducing their impact on amplification. Under Poisson‐based partitioning, each reaction ideally contains either one or no target molecule, which minimizes competition and enables more efficient amplification of low‐copy targets. Together, these effects enhance both sensitivity and robustness, facilitating the detection of low‐abundance targets even in complex environmental samples (Doi et al. 2015; Guri et al. 2024). While qPCR remains a well‐established and efficient method for many ecological applications, the greater sensitivity and ability of dPCR for absolute quantification without the need of standard curves is particularly advantageous for accurately determining gene copy numbers per cell (Bagdonaitė et al. 2025; Doi et al. 2015; Hunter et al. 2017). This not only improves the reliability of converting gene copy numbers into cell abundance estimates but will also be valuable for targeting rare or low abundant taxa, including those exhibiting strong temporal dynamics and seasonality. Digital PCR has increasingly been applied in environmental monitoring of bacteria (Netzer et al. 2021; Ricchi et al. 2017; Wang et al. 2022) and multicellular eukaryotes (Bagdonaitė et al. 2025; Brys et al. 2021; Doi et al. 2015; Maes et al. 2024; Mauvisseau, Davy‐Bowker, et al. 2019; Stelzer et al. 2024). In contrast, its potential for quantitative analyses of protists remains relatively underexplored. This will be especially valuable for monitoring the spatial and temporal distribution of key taxa within interactomes, as they perform important ecological functions and thereby contribute to ecosystem stability (Qu et al. 2020). While previous studies have reported positive correlations between DNA gene copy numbers and species abundance or biomass (Gross et al. 2024; Martin et al. 2022), it is still unclear to what extent dPCR‐derived gene copy numbers can be translated into reliable cell abundance estimates for protists. Establishing whether dPCR can provide reliable abundance estimates for microbial eukaryotes, by thoroughly comparing them with morphological methods, is therefore a critical first step for its broader application in ecological monitoring and quantitative studies of protist communities (Stern et al. 2018).

In this study, we developed and validated species‐specific digital PCR assays for quantifying two planktonic ciliates by comparing dPCR‐derived abundance estimates with classical microscopy counts as a ground truth and with an established CARD‐FISH protocol. Ciliates represent suitable model protists for this evaluation because their relatively high rRNA gene copy numbers facilitate sensitive molecular detection, they can be readily cultivated under controlled laboratory conditions for precise validation experiments, and they are ecologically important components of freshwater plankton communities. Specifically, we first evaluated different primer sets for each target species and developed species‐specific digital PCR assays that convert gene copy numbers into cell abundance estimates using empirically determined gene copy number factors. We then assessed the precision and accuracy of dPCR‐derived abundance estimates against CARD‐FISH counts and evaluated method agreement using controlled laboratory validation experiments. Finally, both methods were applied to environmental field samples from an Austrian lake to compare abundance patterns across spatial, temporal, and environmental gradients. By integrating results from controlled experiments and natural samples, this study provides the groundwork for advancing reliable protist quantification by evaluating the applicability of a novel dPCR approach and examining how methodological differences between molecular and cell‐based approaches may influence ecological interpretations of species distributions. We further assessed under which circumstances dPCR outperforms CARD‐FISH and other microscopy‐based approaches, and how both approaches can be used complementarily.

## 2 Materials and Methods

**FIGURE 1:**
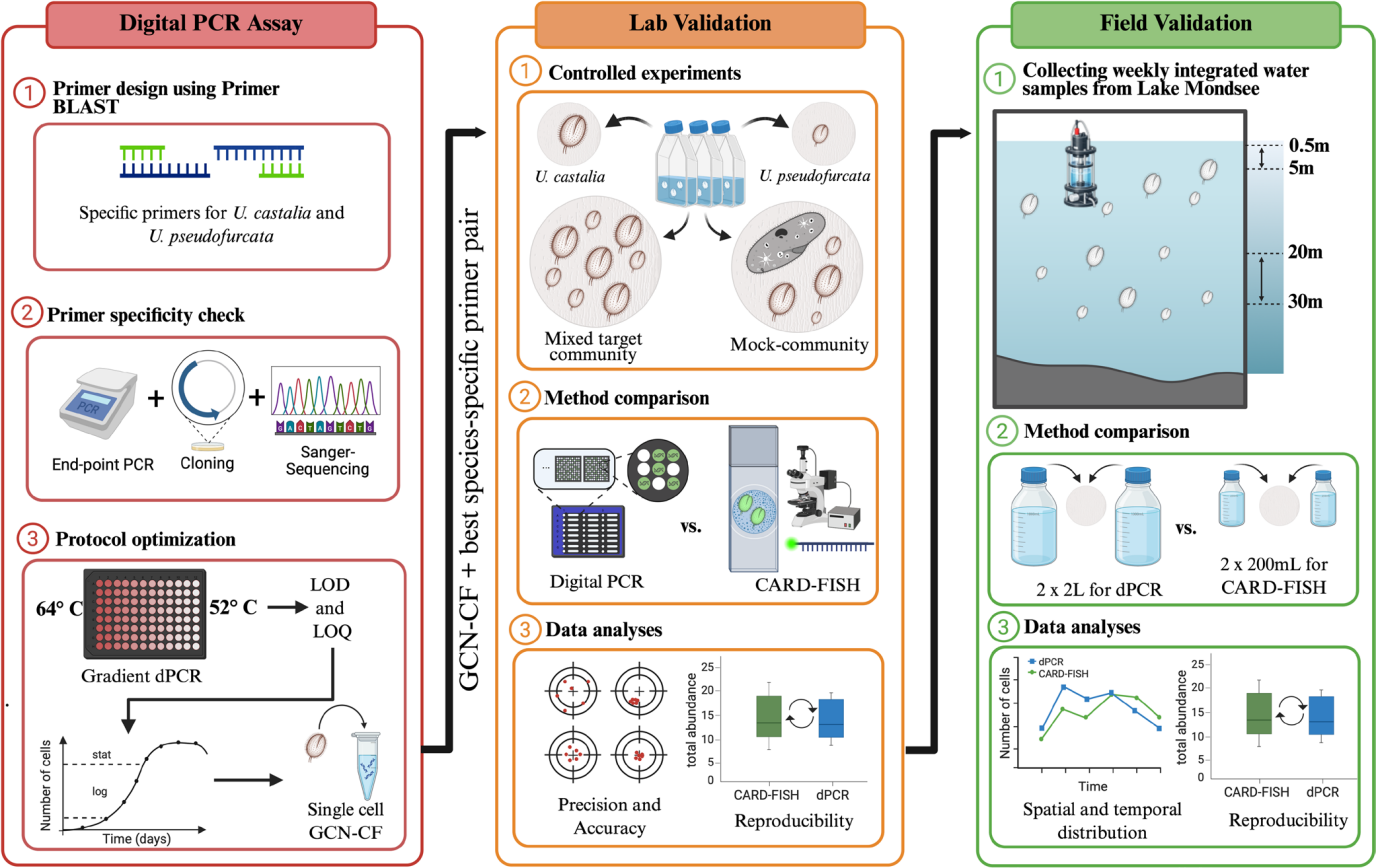
Workflow of the experimental design (created with BioRender).

To validate digital PCR (dPCR) as a quantitative approach for protist abundance estimation, the study followed a hierarchical workflow consisting of three main steps: (1) establishment of a dPCR assay, (2) validation in controlled laboratory experiments in comparison with CARD‐FISH and (3) application to field samples (Figure 1).

### 2.1 Primer Design and Validation

Primers were designed for the two target species *Urotricha castalia* and *Urotricha pseudofurcata* following the requirements for dPCR assays using NCBI's Primer‐BLAST Tool (Whale et al. 2020). We selected the publicly available sequences of the SSU rRNA gene region as targets for our primer design. Although a fragment length of 70–150 bp is recommended for dPCR assays, longer amplicons were required to achieve sufficient primer target specificity. All primer pairs were tested *in silico* on sequences of closely related *Urotricha* species using NCBI‐Primer BLAST to ensure their target specificity. Then, for both species, we evaluated the potential of two different primer pairs (Table 1) and tested them with an initial gradient end‐point PCR (S1.1). For this we combined DNA from environmental samples from Lake Mondsee combined with DNA of *Urotricha castalia*, *Urotricha pseudofurcata*, *Urotricha furcata* and *Urotricha agilis* . PCR‐products were cloned with pGEM‐T Easy Kit (Promega GmbH, Walldorf, Germany) and then grown on agar‐plates according to the manufacturer's protocol. White colonies were selected and plasmids containing the target insert were extracted using the Monarch Plasmid Miniprep Kit (New England Biolabs GmbH, Frankfurt am Main, Germany). Plasmids were then sent for Sanger‐Sequencing using M13 primers (forward: 5′‐CGCCAGGGTTTTCCCAGTCACGAC‐3′/reverse: 5′‐CAGGAAACAGCTATGAC‐3′). The sequencing was performed by StarSEQ GmbH in Mainz, Germany. Primer specificity was evaluated by computing pairwise distance with 1000 bootstraps with reference sequences of the same genus using MEGA v.12.0.1.

**TABLE 1:** Primer pairs for *U. castalia* and *U. pseudofurcata*. Primers were ordered from biomers.net GmbH (Ulm, Germany).

Primer	Target	Sequence (5′‐3′)	Amplicon size (bp = basepairs)
UC833‐1210	*U. castalia*	F: GGCTCTCGTATTGCAAAGCTAGAG R: CCATGCACCACCACCCATAGA	398 bp
UC607‐747	*U. castalia*	F: TGGTGTCGATCTTAGCTGGC R: TCATGACGATTGCCTGCCTT	141 bp
UP1443‐1789	*U. pseudofurcata*	F: GTTTCACCCTGGCCTGGAAA R: TGCTTTACGCACACATACTCCAA	369 bp
UP1596‐1789	*U. pseudofurcata*	F: CCCGTCGCTCCTACCGATTT R: TGCTTTACGCACACATACTCCAA	216 bp

After validation, fivefold dilution series of synthetic oligonucleotides (gBlocks, IDT Technologies; S1.2) representing the respective primer target sequences were analysed to establish the analytical sensitivity of assays. Measures including limit of detection (LOD) and limit of quantification (LOQ) for digital PCR were evaluated and are further described in the Section 2.7.

### 2.2 Controlled Laboratory Experiments and Experimental Sample Processing

#### 2.2.1 Experimental Organisms and Culture Conditions

Laboratory experiments were conducted using cultures of the target organisms *Urotricha castalia* (*U. castalia* strain Cil‐2017/27) and *Urotricha pseudofurcata* (*U. pseudofurcata* strain Cil‐2019/3; Frantal et al. 2022). Non‐target species included *Urotricha agilis* ( *U. agilis* strain Cil‐2017/24), *Urotricha furcata* ( *U. furcata* strain Cil‐2019/5), *Coleps viridis* ( *C. viridis* strain Cil‐2017/13; Pröschold et al. 2021) and *Paramecium bursaria* ( *P. bursaria* strain Cil‐2022/12; Spanner et al. 2022). *Urotricha* spp. and *C. viridis* were maintained in 50 mL culture flasks equipped with ventilation caps (Biomedica, Basel, Switzerland) and grown in a 5:1 mixture of modified Woods Hole MWC medium (Guillard and Lorenzen 1972) and Volvic mineral water. Cultures were incubated at 15°C under a 12:12 h light:dark cycle and fed with *Cryptomonas* sp. strain SAG 26.80 (Culture Collection of Algae, University of Göttingen, Germany; http://sagdb.uni‐goettingen.de). *Paramecium bursaria* was maintained in a 1:5 mixture of modified Woods Hole MWC medium and Volvic mineral water with the addition of a sterile wheat grain.

#### 2.2.2 Single‐Cell Gene Copy Number Estimation

For the evaluation of gene copy number per cell, we followed the experimental setup described in Gross et al. (2024). Briefly, cultures were established in triplicate by inoculating fresh flasks containing WC/Volvic 5:1 medium with *Cryptomonas* sp. strain SAG 26.80 as food source and an initial density of ~20 ciliate cells per flask. Cell abundances were quantified at 24 h intervals by enumerating approximately 10% of each culture using a stereo microscope at up to 45× magnification (Olympus SZ60) to determine exponential and stationary growth phase. Upon reaching the targeted growth phases, single cells were isolated from each culture, washed multiple times in sterile Volvic and subsequently transferred separately into 180 μL of lysis buffer (Qiagen GmbH, Hilden, Germany). Samples were maintained at 4°C until DNA extraction was performed.

#### 2.2.3 Mock Community Assembly

To assess the suitability of dPCR for absolute abundance estimation of both *Urotricha* species and to enable comparison with CARD‐FISH, three dense cultures of each species were selected. Cell concentrations (cells mL^−1^) were first determined by counting 3 × 1 mL replicates per culture. Subsamples were fixed with acidic Lugol's solution (final concentration 5%) and enumerated using a Sedgewick–Rafter counting chamber to determine the culture volume required to obtain defined cell numbers. For each species, appropriate culture volumes were then diluted with sterile WC/Volvic medium (5:1) to final volumes of 10 mL for CARD‐FISH and 50 mL for dPCR, targeting 80, 400, 2000 and 10,000 cells. To account for effects of sample complexity, mock assemblages with different species richness were additionally investigated. First, samples with mixed ratios of *U. castalia* and *U. pseudofurcata* were established in triplicate covering ratios of 1:1 (1000 cells each) and 1:10 (50:500 cells). To increase the complexity, mock assemblages consisting of *U. castalia*, *U. pseudofurcata*, *U. agilis* , *C. viridis* and *P. bursaria* were created in triplicate with 500 cells each (except for *P. bursaria* for which ca. 100 cells were added). *Coleps viridis* and *P. bursaria* both carry green algae as endosymbionts creating an additional complexity. Water samples for dPCR were filtered directly onto Durapore membrane filters (0.65 μm pore size, 45 mm diameter; Millipore Merck KGaA, Darmstadt, Germany) and stored at −20°C until further processing. For CARD‐FISH, samples were preserved with formaldehyde (37% stock; final concentration 1.76%), incubated for at least 1 h at 4°C in the dark and were then gently filtered (< 50 mbar) onto Isopore PC membrane filters (1.2 μm pore size, 25 mm diameter; Merck KGaA, Darmstadt, Germany).

### 2.3 Environmental Sampling and Sample Processing

Samples from Lake Mondsee, Austria, were collected on six sampling dates at weekly intervals between 2 July 2024 and 6 August 2024. On each sampling date, 6.5 L of integrated water samples were taken at the same location from the surface water layer (0.5–5 m depth) and from the deep‐water layer (20–30 m depth) using an Integrated Water Sampler (Hydrobios, Germany). In addition, water temperature profiles, pH, oxygen, chlorophyll, phycoerythrin and conductivity were measured along the water column using a YSI 6600 V2 multi‐parameter probe (Yellow Springs Instruments, USA) and averaged for the respective depth intervals. Collected water samples were pre‐filtered through a 150 μm mesh into a 10 L container to decrease sample complexity, that is, remove larger predatory zooplankton. Containers were stored in cooling boxes and water samples processed immediately after sampling. First, 2 × 200 mL of water were preserved with formaldehyde, incubated at 4°C for at least 1 h in the dark and were then gently filtered onto Isopore PC membrane filters (2.0 μm pore size, 47 mm diameter). For each DNA sample, two biological replicates were generated by pumping ~2 L of water on duplicate filters (Durapore membrane filters, pore size 0.65 μm, diameter 45 mm; Millipore Merck KGaA, Darmstadt, Germany) using a peristaltic pump (flow rate = 50 mL min^−1^). All filters were frozen immediately after filtration at −20°C.

### 2.4 DNA Extraction

DNA from single cells was extracted using the DNeasy Blood and Tissue Kit (Qiagen GmbH, Hilden, Germany) following the manufacturer's instructions, with an adjustment of the final elution volume to 100 μL EB‐buffer as described in Gross et al. (2024).

For laboratory and field analyses, DNA from filter membranes was extracted using the DNeasy PowerWater Kit (Qiagen GmbH, Hilden, Germany) according to the manufacturer's protocol. For samples with higher cell numbers (1000, 2000 and 10,000 cells), the recommended elution volume of 100 μL EB buffer was used. Environmental filters and filters containing lower cell numbers (16, 80 and 400 cells) were eluted using 50 μL EB buffer to increase DNA concentration. After extraction, DNA quality and concentration of environmental samples were determined using a NanoDrop 2000 spectrophotometer (Thermo Fisher Scientific Inc., Waltham, USA). All DNA extracts were stored at −20°C until further analysis.

Prior to DNA extraction, we added an internal positive control (IPC) to our samples to later account for DNA extraction loss. As IPC we used a gBlock fragment of a known concentration (1 × 10^−4^) of a marine bacterial strain (Desulfuromonadaceae; NCBI Accession: JF806801.1). The absence of the strain in our sample was confirmed in initial PCR's using their specific primer set (S1.3).

### 2.5 Digital PCR

Copy number measurements were performed using the QIAcuity One nanoplate‐based digital PCR system (QIAGEN, Germany), which integrates reaction setup, partitioning, amplification, and fluorescence imaging for endpoint PCR readout. Prior to PCR setup, all samples were processed in a dedicated PCR workstation irradiated with UV light for 30 min, and equipment within the workstation was wiped with 80% ethanol to minimize contamination risk.

For dPCR assay optimization a gradient PCR was conducted to determine optimal annealing temperatures before sample analysis. Reactions were run on 96‐well nanoplates (8500 partitions/well) using an annealing temperature gradient of 52°C–64°C (at ∼1.1°C increments) with synthetic oligonucleotide templates (gBlocks Gene Fragments; Intergraded DNA Technologies; Coralville, IA, USA; S1.2). Each 12 μL reaction contained 4 μL QIAcuity EvaGreen Master Mix, 1.2 μL primers (final [0.4 μM]), 1 μL synthetic oligonucleotides, and nuclease‐free water. Cycling conditions were: 95°C for 2 min; 40 cycles of 95°C (15 s), 52°C–64°C (15 s), 72°C (15 s); final hold at 40°C (5 min). Two negative controls (at 61.8°C and 52°C) were included per primer pair.

All subsequent dPCR runs used 24‐well nanoplates (∼26,000 partitions/well) for enhanced sensitivity. Each 40 μL reaction comprised 13.3 μL QIAcuity EvaGreen Master Mix, 4 μL primers (final [0.4 μM]), 0.5 μL EcoRI or XbaI restriction enzyme (0.25 U μL^−1^), template DNA (1 μL for gBlocks, 1–2 μL for environmental samples, 1–9 μL for defined‐cell samples, 20 μL for single cells), and nuclease‐free water. Restriction enzymes were selected by screening each amplicon sequence for potential restriction sites using NEBcutter (v. 3.0) and selecting enzymes that do not cut within the target sequence. Synthetic oligonucleotide dilution series were analysed in 10 technical replicates per dilution level, and all other samples were run in triplicates. Reactions were assembled in standard PCR tubes (Brand, Carl Roth) before loading into nanoplates, which were sealed and cycled as follows: 95°C (2 min); 40 cycles of 95°C (15 s), primer‐specific annealing (15 s), 72°C (15 s); 40°C hold (5 min). Three non‐template controls (nuclease‐free water) were included per plate.

Post‐amplification, nanoplates were imaged in the green channel (EvaGreen signal) with 150 ms exposure and gain 6. Relative fluorescence unit (RFU) thresholds were kept as automatically calculated by the software to classify partitions as positive or negative.

### 2.6 Card‐Fish

For both *Urotricha* strains, species‐specific oligonucleotide probes from Dirren‐Pitsch et al. (2022) were selected: Uro2‐1440 (Sequence: 5′‐CGTTGACTCAAGGACAACGACGGTCCAG‐3′) for *U. castalia* and Uro5‐403 (Sequence: 5′‐TGAAAGGACCCCGAGTTGTT‐3′) for *U. pseudofurcata*. CARD‐FISH followed the protocol of Dirren‐Pitsch et al. (2022), which we describe here briefly: After sample filtration, all filters were embedded in 0.1% agarose to minimize detachment of cells, dried at 37°C on parafilm for 30 min, and treated with 0.01 M HCl for 20 min to suppress endogenous peroxidase activity. For each filter and probe, three technical replicates (1/12 of filter) were subjected to the CARD‐FISH protocol. Filters were hybridized with HRP‐labelled probes under probe‐specific conditions (see Dirren‐Pitsch et al. 2022). Signal amplification was achieved using fluorochrome‐labelled tyramides, followed by DAPI counterstaining. To reduce background signal, only 1 μL of tyramide solution mixture was used. Filter sections were mounted on glass slides and stored at −20°C until evaluation. A detailed list of chemicals is provided in S1.4 and buffer preparation protocols followed Dirren‐Pitsch et al. (2022).

All slides were investigated using the Axiostar plus microscope (Carl Zeiss AG, Oberkochen, Germany) equipped with a HXP 120 mercury lamp. For visualization of cells, two different filter sets were used: Filter set Zeiss 02 was used for UV excitation (excitation: G 365 nm, beam splitter: FT 395, emission: LP 420) to visualize DAPI‐stained nuclei and filter set Zeiss 09 (excitation: BP 450–490, beam splitter: FT 510, emission: LP 515) to detect fluorescein isothiocyanate (FITC) labelled cells. Evaluation of filters was performed at 100×–1000× magnification by investigating triplicate filter sections (1/4 of the total filter) for each target. High‐resolution images were taken with the microscope Axio Observer. Z1 using the software Zen v.3.10 to visualize stained nuclei, autofluorescence of algae and FITC‐labelled cells. Overlays were created with the software Fiji v.1.54p (Schindelin et al. 2012).

### 2.7 Data Analyses

All statistical analyses were performed in R (v.4.3.1) using the packages tidyverse (v.2.0.0), scales (v.1.3.0), car (v.3.1–2), rstatix (v.0.7.2), ggpubr (v.0.6.0), knitr (v.1.45), DescTools (v.0.99.57), patchwork (v.1.2.0), lmerTest (v.3.1–3), emmeans (v.1.11.1) and performance (v.0.15.0). Graphical outputs were generated using ggplot2 (v.3.5.1). A detailed list of all packages used for analyses and creating figures is provided in the R code that can found here: https://doi.org/10.5281/zenodo.19147375.

To assess the analytical sensitivity of the dPCR assays, limits of detection (LOD) and quantification (LOQ) were determined for each primer set specific to *U. castalia* and *U. pseudofurcata*. The methodology is described in detail in Gross et al. (2025) and in the accompanying R code. LOD and LOQ were estimated using serial fivefold dilutions of respective synthetic oligonucleotides. DNA concentrations were quantified with a Quantus fluorometer using the QuantiFluor One dsDNA dye (Promega, Walldorf, Germany), and expected gene copy numbers were calculated as described in Gross et al. (2025).

#### 2.7.1 Gene Copy Number Correction

To establish species‐specific gene copy number correction factors (GCN‐CFs), gene copy numbers per cell were compared between exponential and stationary growth phases for each target species. After evaluating normality and homogeneity of variance, phase‐specific differences were assessed using two‐sample *t*‐tests to confirm that growth phase did not influence gene copy numbers and that mean gene copy numbers per species could be used as correction factors. IPC correction factors were derived by normalizing the expected gene copy number of the original gBlock to the gene copy number recovered after spiking into a DNA sample, subsequent extraction, and quantification by dPCR. Absolute cell abundances were then calculated as:

(1) $${\text{Cells}}_{\text{total}}=\left({\mathrm{GCN}}_{\mathrm{\mu L}\;\mathrm{DNA}}\times V_{\mathrm{ext}}\times {\mathrm{IPC}}_{\mathrm{cor}}\right)/{\mathrm{GCN}}_{\mathrm{CF}}$$

where Cells_total_ represents the estimated number of cells in the processes sample, GC_μL DNA_ is the number of gene copies measured per μL of DNA template by dPCR, V_ext_ is the total DNA extraction volume of the respective sample, IPC_cor_ is the extraction efficiency correction factor derived from the internal positive control (IPC), and GCN_CF_ is the species‐specific gene copy number correction factor representing the average number of target gene copies per cell.

#### 2.7.2 Evaluating Method Performance

The performance of the dPCR assays was evaluated by comparing abundance estimates derived from dPCR and CARD‐FISH with each other and with expected cell numbers obtained from initial Sedgewick rafter chamber counts. Prior to statistical analyses, abundance values were log‐transformed to reduce overdispersion and stabilize variance. Method precision was evaluated by calculating the coefficient of variation (CV) between technical replicates and method accuracy was assessed by comparing observed abundance estimates with expected cell numbers and calculating the relative error of each. Workflow stability was evaluated by calculating CV between independent reaction or filter replicates for controlled experiments. To test whether precision and accuracy differed between methods while accounting for variation in expected abundance and sample complexity, linear mixed‐effects models were applied with method as a fixed effect and sample identity included as a random effect to account for paired measurements. We further assessed the agreement between dPCR and CARD‐FISH by applying different complementary measures including regression analyses, the Concordance Correlation Coefficient (CCC), and Bland–Altman analyses.

Differences in abundance estimates of environmental samples between methods across depth and sampling dates were evaluated using linear mixed‐effect models including method, depth, and sampling date as fixed effects and sample identity as a random effect. The environmental variables we measured in the water column were highly collinear and therefore summarized using principal component analysis (PCA) with the first two components explaining 88.5% of the variation. Since the first component primarily reflected vertical stratification (temperature, conductivity and pH), only the second component, mainly associated with chlorophyll and phycocyanin (and to a lesser extent oxygen and conductivity), was retained in environmental response models to avoid redundancy.

## 3 Results

### 3.1 Species‐Specific dPCR Assays

All primer pairs demonstrated high target specificity, as confirmed by similarity matrices of Sanger‐sequenced amplicons (S1.5–8). Gradient dPCR assays showed clear separation between positive and negative partitions across most tested annealing temperatures (S1.9–10), with minimal intermediate ‘rain’. An annealing temperature of 58.5°C provided consistent discrimination of partitions and amplification efficiency across all primer sets and was therefore selected as a common temperature to enable multiplexed plate runs.

**FIGURE 2:**
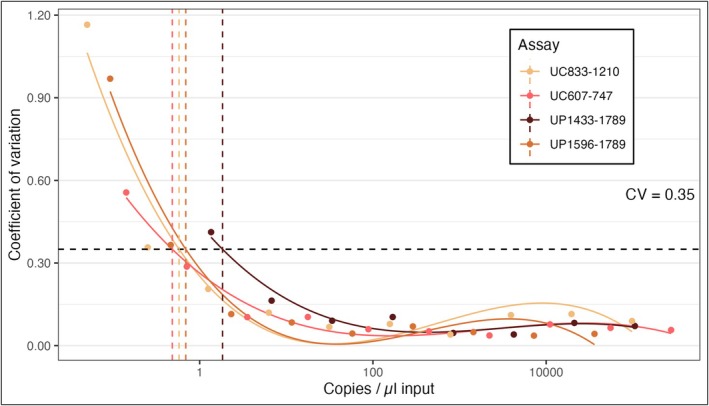
Limit of quantification (LOQ) for UC833‐1210, UC607‐747, UP1433‐1789 and UP1597‐1789. The dashed black horizontal line represents the 0.35 coefficient of variation (CV) that was used to calculate the LOQ. Dashed vertical lines represent the LOQ values of UC833‐1210 (0.578, yellow), UC607‐747 (0.482, pink), UP1433‐1789 (1.835, dark brown) and UP1597‐1789 (0.691, light brown).

Subsequent serial dilution series revealed performance differences among primer sets in terms of precision and quantification range. Limit of detection (LOD) was estimated at 0.048 copies/μL for *U. castalia* specific primer sets UC833‐1210 and 0.146 copies μL^−1^ for UC607‐747 (S1.11–12). Although UC833‐1210 showed a lower LOD, UC607‐747 demonstrated superior linearity (*β*_1_ = 1.026, 95% CI: 1.022–1.029) and a lower limit of quantification (LOQ) (0.482 vs. 0.578 copies μL^−1^ for UC833‐1210, Figure 2), yielding better overall performance. Similarly, *U. pseudofurcata* specific primer sets indicated that UP1596‐1789 outperformed UP1433‐1789, demonstrating greater linearity (*β*_1_ = 1.002, 95% CI: 0.999–1.005 vs. *β*_1_ = 1.067, 95% CI: 1.064–1.071) and substantially lower detection and quantification limits (LOD = 0.074 vs. 1.741 copies μL^−1^; LOQ = 0.691 vs. 1.835 copies μL^−1^, S1.13–14), enabling reliable quantification across the full concentration range (Figure 2). Both UC607‐747 and UP1596‐1789 were therefore selected for downstream analyses.

No significant difference was detected between the exponential and stationary growth phases for *U. castalia* (Welch's *t*‐test: *t*_38.34_ = −0.53, *p* = 0.60) or *U. pseudofurcata* (two‐sample *t*‐test on log‐transformed values: *t*_34_ = −0.34, *p* = 0.73; S1.15). Data were therefore pooled, yielding overall means of 1852 ± 790 copies cell^−1^ for *U. castalia* and 699 ± 422 copies cell^−1^ for *U. pseudofurcata*, which were then applied as a gene copy number correction factor (GCN‐CF) for each target.

Measured IPC gene copy numbers indicated moderate variability in extraction recovery and were on average 11.51‐fold lower than direct dPCR quantification of the same input, corresponding to an extraction recovery of approximately 8.7% (S1.16). This correction factor was incorporated together with the GCN‐CF into Equation (1) (see Section 2.7) to adjust absolute cell abundance estimates. Based on these correction factors and the estimated LOQ values, the theoretical minimum quantifiable abundance corresponded to fewer than 5 cells per sample for both target species.

### 3.2 Laboratory Validation Using Controlled Experiments

**FIGURE 3:**
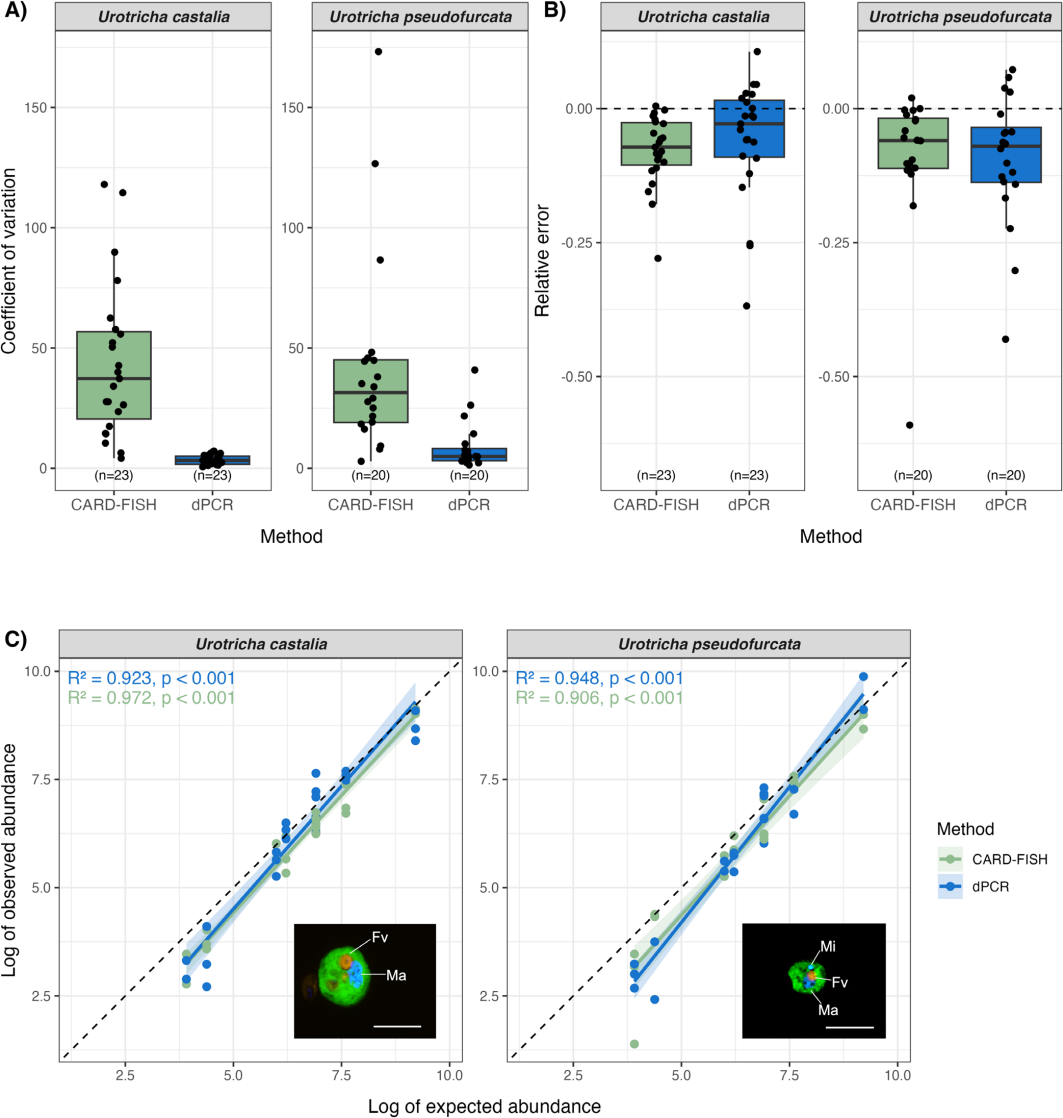
Performance comparison of CARD‐FISH and digital PCR (dPCR) for quantifying *U. castalia* and *U pseudofurcata* in laboratory validation experiments. (A) Coefficient of variation (CV) among technical replicates for CARD‐FISH (green) and dPCR (blue). Points represent sample‐level estimates; boxplots show medians and interquartile ranges. (B) Relative error between observed and expected abundances (log‐scale proportional deviation). The dashed horizontal line indicates perfect agreement (relative error = 0). (C) Relationship between log‐transformed expected and observed abundances. Individual datapoints show workflow replicates and solid lines show linear regressions with 95% confidence intervals (shaded area); the dashed line indicates 1:1 agreement. Adjusted *R* ^2^ and *p*‐values are shown for each regression. Microphotographs show overlays of Z‐stack images for *U. castalia* using probe Uro2‐1440 and *U. pseudofurcata* using probe Uro5‐403. Fv = food vacuole; Ma = macronucleus; Mi = micronucleus. Scale bars = 20 μm.

All CARD‐FISH probes demonstrated high target specificity in controlled experiments (Figure 3) and were empirically confirmed by the absence of cross‐hybridization with non‐target taxa in mixed communities (S1.17). Hybridized cells exhibited a strong and consistent fluorescent signal following tyramide signal amplification, which was clearly distinguishable from background fluorescence and from non‐target cells. No unspecific staining or ambiguous signal patterns were observed across the tested abundance range.

Comparison of method precision, quantified as the coefficient of variation (CV) among technical replicates, revealed pronounced differences between dPCR and CARD‐FISH for both targets in controlled experiments (Figure 3A). Mean technical CV for dPCR was 3.5% ± 2% for *U. castalia* and 8.8% ± 10% for *U. pseudofurcata*, whereas CARD‐FISH exhibited substantially higher variability (43.7% ± 32% and 42.7% ± 42%, respectively). Linear mixed models confirmed a significant effect of method on technical precision for both species (*U. castalia*: *β* = −1.10 ± 0.37 SE, *t*₄₀ = −2.99, *p* = 0.0048; *U. pseudofurcata*: *β* = −1.12 ± 0.45 SE, *t*_34_ = −2.47, *p* = 0.019; S1.18), with dPCR exhibiting approximately an order‐of‐magnitude lower CV than CARD‐FISH across the tested abundance range. Technical variability decreased significantly with increasing expected abundance for both methods (*U. castalia*: *β* = −0.30 ± 0.09 SE, *p* = 0.001; *U. pseudofurcata*: *β* = −0.41 ± 0.11 SE, *p* < 0.001), whereas species richness (community complexity) had no detectable effect. No significant method × abundance or method × richness interactions were observed (S1.18).

In contrast to technical precision, workflow‐level variability across independent extractions/filters of the same controlled experiment was comparable between methods. Median CV ranged between 23%–30% across species and methods, with no consistent difference between them (S1.19). However, interquartile range revealed that workflow variability was more consistent for dPCR in *U. castalia*, whereas variability was highly heterogeneous across samples for both methods in *U. pseudofurcata* (S1.19). Linear mixed models confirmed the absence of a significant overall method effect for *U. castalia* (*β* = 0.17 ± 0.87 SE, *t*₈ = 0.20, *p* = 0.85; S1.20). For *U. pseudofurcata*, no significant main effect of method was detected (*β* = 2.21 ± 1.10 SE, *t*₈ = 2.01, *p* = 0.079). Although a significant method × richness interaction was observed for *U. pseudofurcata* (*β* = −1.00 ± 0.40 SE, *t*₈ = −2.48, *p* = 0.038; S1.20), this effect should be interpreted cautiously given the limited sample size.

Analysis of relative error, calculated as the log‐scale proportional deviation between observed and expected abundances, generally indicated small but comparable deviations for both methods and species (Figure 3B). For *U. castalia*, both CARD‐FISH and dPCR showed slight underestimation of expected abundances, and deviations for *U. pseudofurcata* were similarly modest and centred close to zero. Linear mixed models confirmed the absence of a significant method effect on relative error for either species (*U. castalia*: *β* = 0.053, *p* = 0.87; *U. pseudofurcata*: *β* = −0.195, *p* = 0.58; S1.21), indicating comparable quantitative accuracy. However, intercept estimates were significantly negative for both species, suggesting a slight overall underestimation across methods (*U. castalia*: *β* = −0.609, *p* = 0.034; *U. pseudofurcata*: *β* = −0.679, *p* = 0.010; S1.21). Regression analyses further demonstrated strong agreement between observed and expected abundances across the tested concentration range (Figure 3C). For *U. castalia*, observed cell numbers were highly correlated with expected values for both CARD‐FISH (*R* ^2^ = 0.972, *p* < 0.001) and dPCR (*R* ^2^ = 0.923, *p* < 0.001). Similarly, for *U. pseudofurcata*, strong linear relationships were detected for CARD‐FISH (*R* ^2^ = 0.906, *p* < 0.001) and dPCR (*R* ^2^ = 0.948, *p* < 0.001). Regression slopes closely followed the 1:1 expectation, indicating robust quantification across the full abundance gradient.

Linear mixed‐effects models showed no effect of community complexity (richness) on accuracy for either *U. castalia* or *U. pseudofurcata* (S1.21). Similarly, no method × richness or method × abundance interaction effect was observed. For *U. pseudofurcata*, relative error was significantly influenced by expected abundance, indicating improved accuracy at higher cell concentrations for both methods.

**FIGURE 4:**
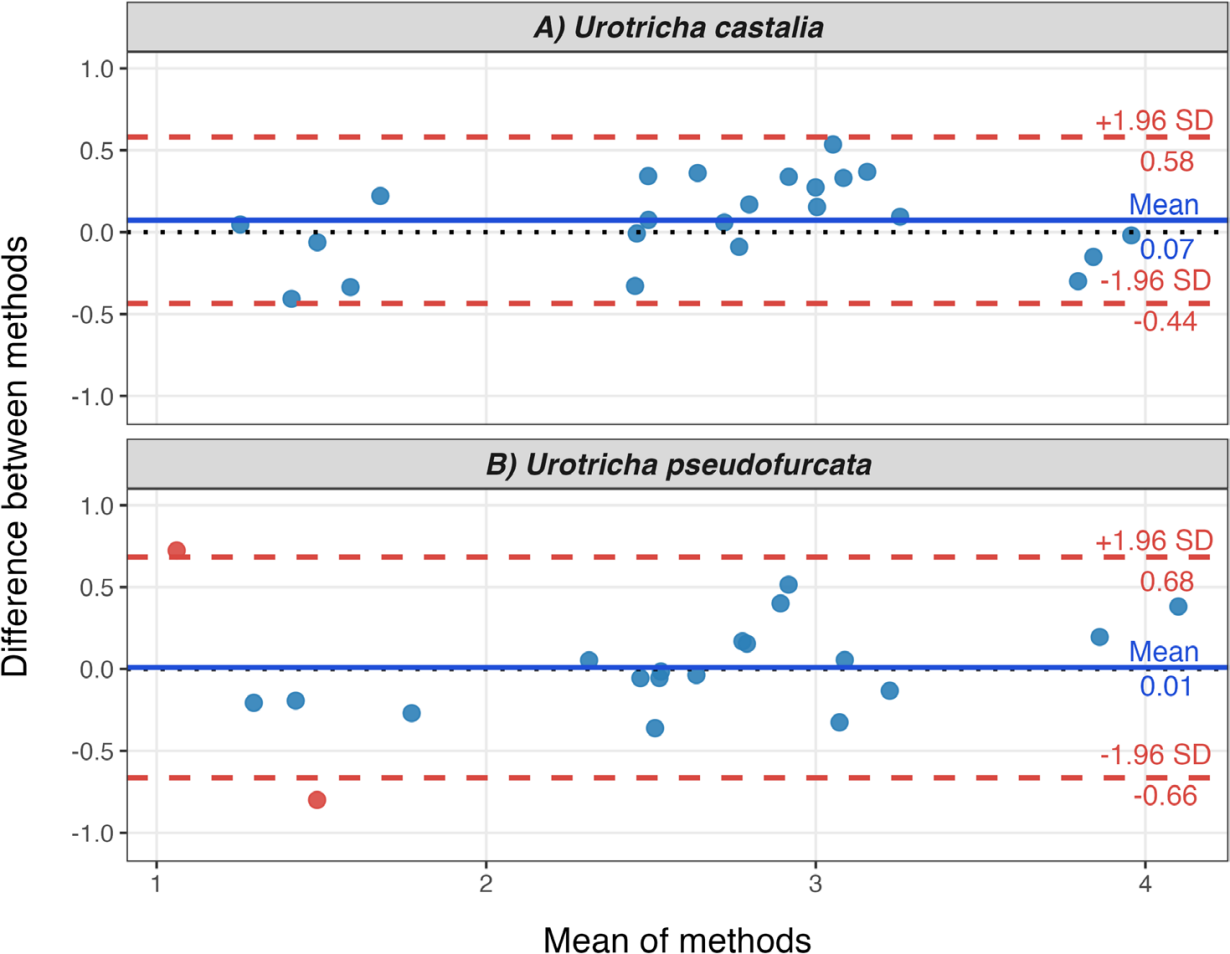
Bland–Altman plots illustrating agreement between dPCR and CARD‐FISH for *U. castalia* (A) and *U. pseudofurcata* (B) under controlled experimental conditions. Differences between methods were calculated as abundance (dPCR) – abundance (CARD‐FISH). Abundance data were log transformed prior to analysis to reduce heteroscedasticity. Each point represents and individual sample. The solid blue line represents mean bias, and the dashed red line denotes the 95% limits of agreement (mean difference ±1.96 × SD). Point colours indicate whether observations fall within (blue) or outside (red) the limit of agreement.

Reproducibility between dPCR and CARD‐FISH in controlled experiments was further evaluated using Bland–Altman analyses. A minimal mean positive difference was observed indicating that dPCR yielded marginally higher abundance estimates than CARD‐FISH for *U. castalia* and *U. pseudofurcata* (0.07 and 0.08 log units, respectively; Figure 4A). No evidence of proportional bias was detected for either species (*U. castalia*: *β*_1_ = 0.073, *p* > 0.05; *U. pseudofurcata*: *β*_1_ = 0.120, *p* > 0.05). Limits of agreement indicated that most differences fell within approximately ±0.5–0.7 log units (Figure 4B), demonstrating strong overall agreement across the tested abundance range. Concordance between methods was further supported by high Lin's concordance correlation coefficients (CCC = 0.94, 95% CI: 0.87–0.97 for *U. castalia*; CCC = 0.91, 95% CI: 0.80–0.96 for *U. pseudofurcata*).

### 3.3 Field Validation Using Environmental Samples From Lake Mondsee

**FIGURE 5:**
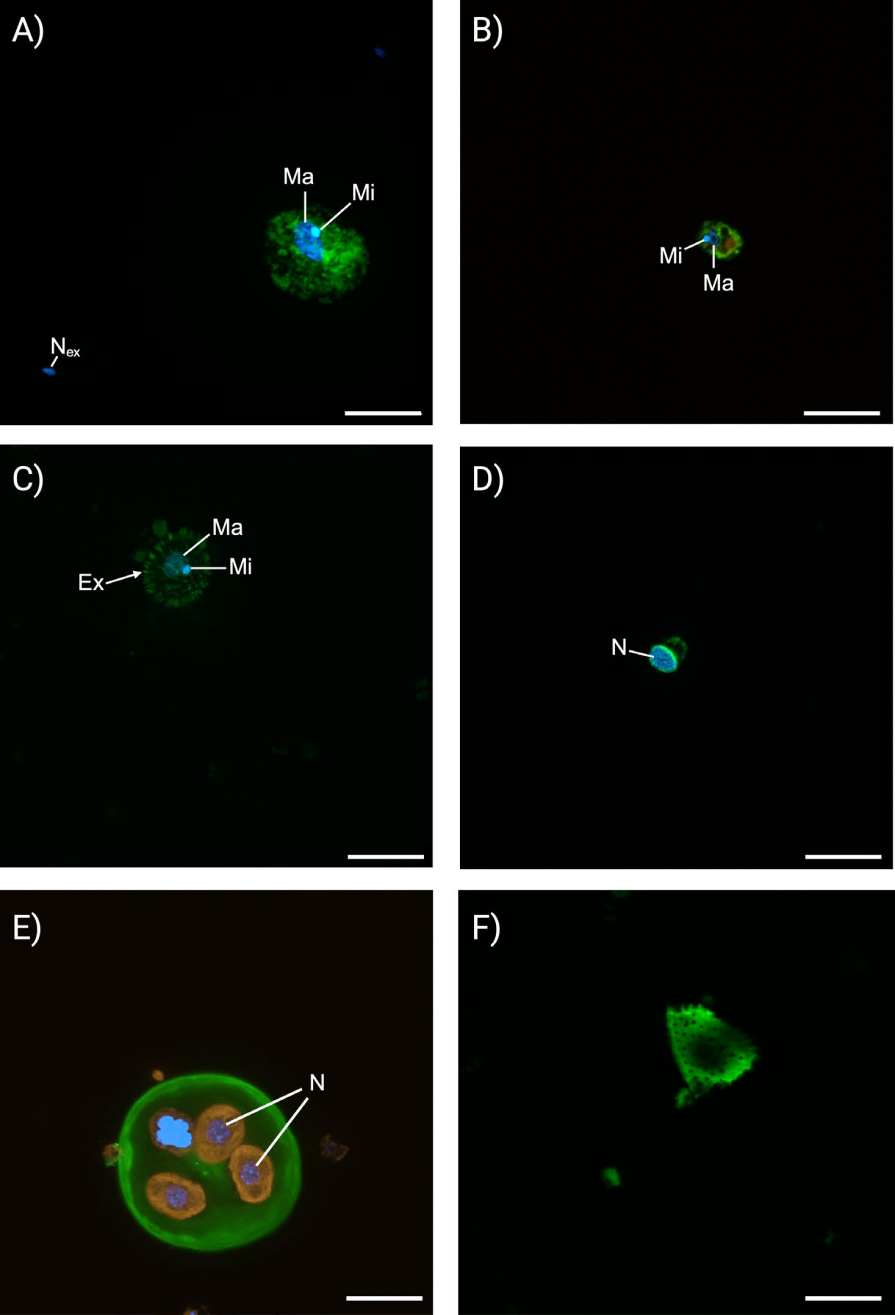
Microphotographs of CARD‐FISH from environmental samples. Images show overlays using combined filter sets for DAPI stained nuclei (blue), hybridization signal (green) and chlorophyll *a* autofluorescence (red) using probes Uro2‐1440 (A, C, E) and Uro5‐403 (B, D, F). (A) Hybridized *U. castalia*, (B) hybridized *U. pseudofurcata*, (C) false‐positive signal from extrusomes of ciliate, (D) false‐positive signal for another? ciliate species, (E) false‐positive signal for unidentified protist, and (F) false‐positive signal for cell structure probably of a dinoflagellate. Scale bars are 20 μm. Ex = extrusomes, Fv = Food vacuole, Ma = macronucleus, Mi = micronucleus, N = nucleus/nuclei, N_ex_ = extracellular nuclei.

In environmental samples, dPCR showed quantifiable detections for all 24 samples (100%) for *U. castalia* and for 22 of 24 samples (92%) for *U. pseudofurcata*, whereas CARD‐FISH detected the same taxa in 11 of 24 (46%) and 19 of 24 (79%) samples, respectively. All dPCR abundances are reported as estimated cell concentrations (cells L^−1^), corrected for species‐specific rRNA gene copy number and extraction efficiency as described above. CARD‐FISH probes produced clear signals for the target organisms (Figure 5A,B). However, additional fluorescence was occasionally observed from non‐target structures (e.g., extrusomes and food of ciliates, probably dinoflagellate cortex structures, etc.; Figure 5C–F). Despite the treatment of filters with HCL to inactivate endogenous peroxidases, we frequently observed a fluorescent signal emitted from extrusomes (Figure 5C).

**FIGURE 6:**
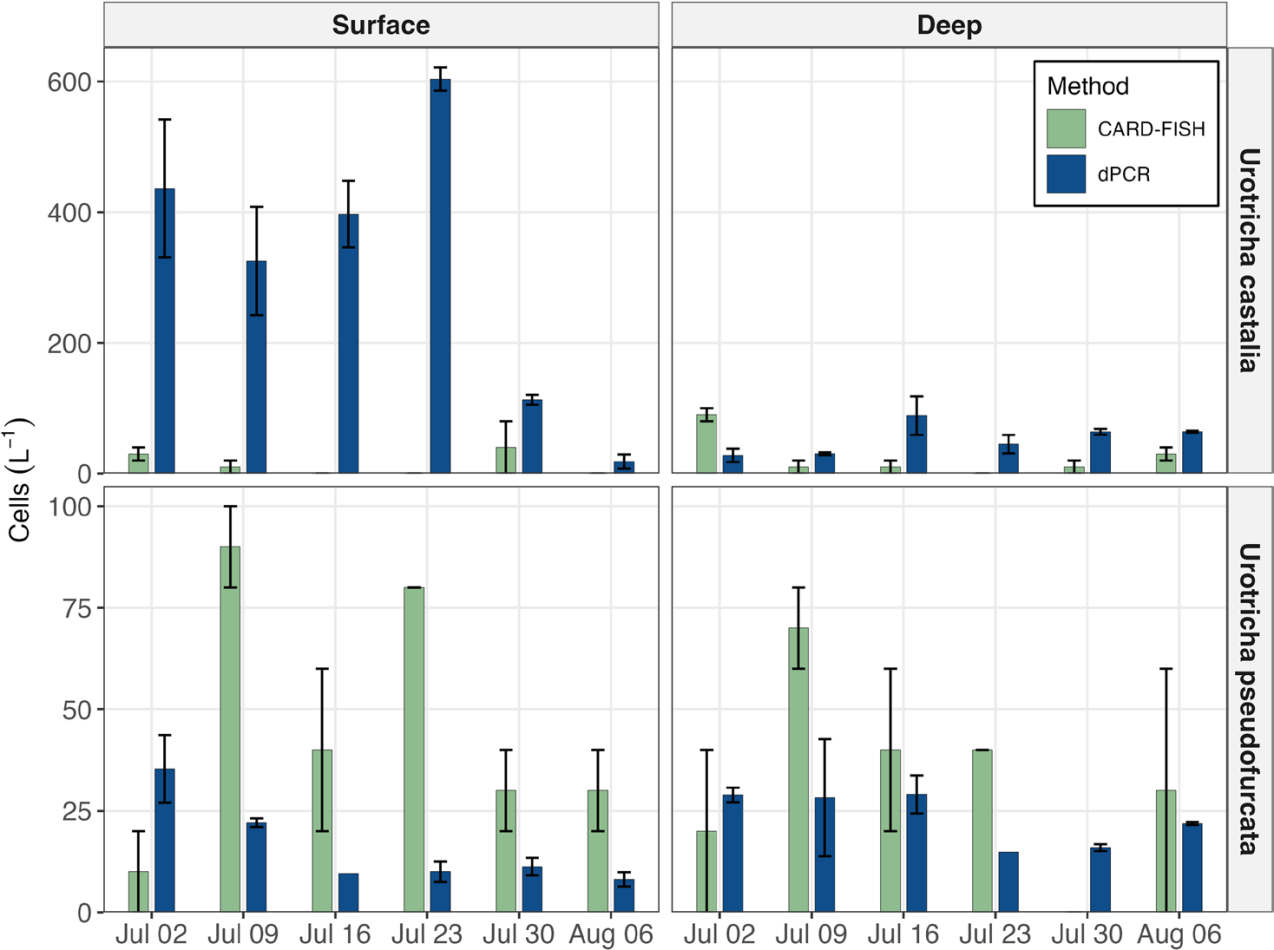
Field validation of dPCR and CARD‐FISH for quantifying cell abundances (cells L^−1^) in water samples collected from Lake Mondsee during summer 2024 (July–August). Upper panels show abundance patterns of *U. castalia*, and lower panels show those of *U. pseudofurcata*. Samples were obtained from two depth strata: Surface (integrated samples from 0.5 to 5 m) and deep (integrated samples from 20 to 30 m). Bars represent mean values from two biological replicates (*n* = 2), and error bars indicate ± standard error.

In surface waters, mean abundance of *U. castalia* across all sampling days was 316 ± 215 cells L^−1^ for dPCR and 13 ± 25 cells L^−1^ for CARD‐FISH, whereas in deeper layers mean abundance was 53 ± 27 and 25 ± 33 cells L^−1^, respectively. For *U. pseudofurcata*, mean abundance was 17 ± 11 (dPCR) and 47 ± 32 cells L^−1^ (CARD‐FISH) at the surface, compared to 24 ± 9 and 33 ± 29 cells L^−1^ in deeper layers. A clear discrepancy between methods was observed for *U. castalia* for both depths (Figure 6). For *U. castalia*, dPCR yielded significantly higher abundance estimates than CARD‐FISH (*β* = 1.78 ± 0.24 SE, *t*_34_ = 7.46, *p* < 0.001), corresponding to an approximately 24‐fold higher abundance. This was especially pronounced in surface waters, indicating that vertical structuring was not consistently captured by both approaches (method × depth: *F*(1, 34) = 8.68, *p* = 0.006; S1.22). By contrast, for *U. pseudofurcata* no significant difference in abundance pattern was observed between both methods (*β* = −0.31 ± 0.21 SE, *t*_34_ = −1.5, *p* = 0.14) and differences in vertical patterns were negligible (method × depth: *F*(1, 37) = 3.89, *p* = 0.056; S1.22).

Across both methods, linear mixed‐effects models revealed no significant day‐to‐day variation in *U. castalia* abundance (*F*(5, 5) = 0.87, *p* = 0.56), whereas *U. pseudofurcata* showed weak evidence of temporal dynamics (*F*(5, 37) = 2.21, *p* = 0.074). In addition to depth and temporal variation, we explored whether broader environmental gradients (summarized via PCA) influenced abundance patterns and whether such responses differed between methods. For *U. pseudofurcata*, the second principal component exhibited a significant method‐dependent effect (method × PCenv2: *F*(1, 34) = 7.48, *p* = 0.010). Trend estimates indicated a positive association between CARD‐FISH–derived abundance and PCenv2 (*β* = 0.244 ± 0.078 SE), whereas no association was detected for dPCR (slope ≈0). When models were fitted separately for each method, residual standard deviation was lower for dPCR (0.53 log units) than for CARD‐FISH (0.85 log units), indicating greater unexplained variability in CARD‐FISH–derived abundance estimates.

## 4 Discussion

This study underscores the importance of systematically comparing methodological approaches to improve abundance estimates of protists and demonstrates the potential of digital PCR (dPCR) for quantitative applications. We successfully validated dPCR assays for two planktonic ciliates using newly designed species‐specific primers and established gene copy number correction factors. In controlled laboratory experiments, quantification by dPCR and CARD‐FISH was reproducible and showed comparable accuracy, while dPCR exhibited higher precision. In field samples, however, dPCR showed enhanced sensitivity compared to CARD‐FISH, and methods revealed species‐specific differences in spatial and temporal patterns. These observations emphasize the potential of dPCR as a high‐throughput and sensitive quantification method for ciliates and potentially other protists in environmental samples. We consider dPCR to be especially promising for long‐term monitoring of key taxa involved in ecological interactions in the context of climate change and other disturbances and for targeting rare or low‐abundance taxa.

### 4.1 Improving dPCR‐Based Abundance Estimates

For both target species, we successfully developed highly sensitive dPCR assays. The clear separation of positive and negative partitions across primer sets indicated robust assay performance. Estimated LOD and LOQ values showed that even very low amounts of target DNA could be detected and quantified, theoretically corresponding to fewer than 10 cells. Although these limits were determined using synthetic oligonucleotides, numerous studies have similarly reported high sensitivity of dPCR platforms and low detection thresholds for a variety of organisms, including ciliates (Gross et al. 2025; Hunter et al. 2017; Mauvisseau, Burian, et al. 2019; Stelzer et al. 2024), highlighting the potential of dPCR to detect rare or low abundant organisms. We also observed that the introduction of IPCs before DNA extraction greatly improved our abundance estimates. Many studies have already pointed out that DNA loss can be substantial and recovery could often be reduced to less than 5% (Zöhrer et al. 2025). We found similar results with recovery being only 8.7% of the expected copies. Incorporating IPCs, especially in environmental dPCR studies, has already demonstrated great improvements for other studies capturing eDNA (Brys et al. 2021; Everts et al. 2021; Stelzer et al. 2024). We therefore strongly recommend applying IPCs to increase quantification accuracy. We further want to point out that when designing primers, the size of the amplified product needs to be considered carefully. Although primers targeting smaller amplicons resulted in improved LOD and LOQ, they may also be associated with reduced recovery rates, which could influence quantification outcomes (Zöhrer et al. 2025).

rRNA gene copy numbers per cell can vary substantially within protist species depending on their physiological state or cell cycle stage (Gong et al. 2013; Gross et al. 2024; Wang et al. 2019, 2020). Although we found considerable variation in both ciliates, we did not observe a clear association with growth phase under the conditions tested here, as was observed elsewhere (Fu and Gong 2017). Further work on the influence of growth phases together with different temperatures and nutritional modes is needed to clarify their relationship with gene copy numbers. Because multiple single cells were analysed with biological and technical replication, we consider our estimates robust and attribute the remaining variation to other confounding factors not explored here but discussed elsewhere (Lavrinienko et al. 2021). Both investigated species belong to the genus *Urotricha* but morphologically differentiate considerably in size, that is, *U. pseudofurcata* measures 16–24 × 10–19 μm and *U. castalia* 38–59 × 25–44 μm (Frantal et al. 2022). Consistent with previous studies (Fu and Gong 2017; Girard et al. 2024; Liu et al. 2021; Vasselon et al. 2018), we observed a positive association between gene copy number per cell and cell size, as average values for *U. pseudofurcata* were approximately 2.5 times lower than those of *U. castalia*. These differences may also reflect variation in micro‐ and macronuclear size, which determine total DNA content and thus gene copy numbers, as discussed previously (Frantal et al. 2022; Stoeck et al. 2014). Together, these findings suggest that the establishment of group‐specific GCN‐CF for ciliates should account for organism as well as nuclear size, as previously proposed for other protist groups (Girard et al. 2024). While not being the primary objective of this study, the rRNA gene copy numbers determined here for two common and ecologically important ciliate species help fill existing knowledge gaps and may contribute to the development of reference database supporting quantitative interpretations of future metabarcoding studies as already suggested by Stoeck et al. (2014).

It has been an ongoing discussion whether absolute quantification of protists can be realized from molecular data given their extensive inter‐ and intraspecific variation in rRNA gene copy numbers with some cells harbouring several thousand copies (Lavrinienko et al. 2021). Improving molecular quantification therefore requires systematic comparisons between emerging and established methods. While many studies have compared morphospecies counts with rDNA read abundances and reported inconsistencies (Frantal et al. 2022; Pitsch et al. 2019; Pröschold et al. 2021; Stoeck et al. 2014), the use of correction factors has rarely been explored. In our controlled laboratory experiments, applying species‐specific GCN‐CF together with extraction efficiency corrections substantially improved dPCR‐based abundance estimates and increased agreement with CARD‐FISH. Similar improvements were reported by other studies who achieved better correspondence between quantitative metabarcoding data based on rDNA reads and morphospecies counts after applying group‐specific GCN corrections (Girard et al. 2024; Martin et al. 2022). Together, these findings highlight the potential of incorporating correction factors to refine molecular quantification approaches.

### 4.2 Comparative Precision and Accuracy of dPCR and CARD‐FISH Derived Abundance Estimates

For controlled experiments, we surprisingly observed consistent results across both methods. While we initially expected CARD‐FISH to provide more accurate estimates, our correction factors reduced relative error of the dPCR‐derived abundance estimates to the level of CARD‐FISH. Despite the improved accuracy with GCN‐CF, we nonetheless observed a general underestimation of abundances for both methods. Digital PCR has been shown to provide highly precise and accurate estimates of DNA copy number concentrations, with reported measurement uncertainties below 5% across a wide dynamic range (Pinheiro et al. 2012). Deviations between gene copy numbers and cell abundances are therefore more likely attributable to biological variability in rRNA gene copy numbers per cell rather than inaccuracies by the dPCR quantification itself. CARD‐FISH underestimations likely arise from incomplete recovery of intact cells during filtration and hybridization, as fragile protists may be disrupted during fixation or lost during processing, reducing the effective capture probability of target cells (Dirren‐Pitsch et al. 2022; Pfister et al. 1999; Piwosz et al. 2021).

When investigating technical precision between methods, we found strong method‐dependent differences with dPCR outperforming CARD‐FISH. While CARD‐FISH largely eliminates identification bias by using species‐specific probes, technical variability may still arise from counting statistics and subsampling of filter areas, particularly when target organisms occur at low abundances. By contrast, in dPCR random error causing imprecise quantification is likely reduced due to the partitioning of samples (Hunter et al. 2017; Wood et al. 2019). However, for both methods, we observed a significant influence of target concentration, with increased repeatability among technical replicates at higher abundances. Similar concentration‐dependent precision has been reported for dPCR in a previous study (Gross et al. 2025). The pattern observed for CARD‐FISH in our experiments likely reflects the greater stochastic variability associated with detecting and counting rare targets. These results suggest that particularly for low‐abundant targets, dPCR may provide higher precision.

Environmental studies have increasingly raised concerns that workflows may not reliably reproduce quantification results for target species (Mauvisseau, Burian, et al. 2019; Pollock et al. 2018; Sepulveda et al. 2020). In our controlled experiments, we observed moderate variability between workflow replicates for both approaches. Although overall variability was comparable between methods, dPCR produced more consistent results across samples, indicating higher reproducibility, whereas CARD‐FISH showed greater variability likely related to sample processing. While some variations in dPCR may result from biological variation in rRNA gene copies and DNA extraction efficiency, variation in CARD‐FISH likely reflects a heterogeneous cell distribution on filters and a stochastic cell loss during filtration or hybridization steps. To minimize the influence of such variability, we recommend including multiple independent replicates when quantifying target species, regardless of the method applied.

### 4.3 Method‐Dependent Differences in Environmental Abundance Patterns

In contrast to the controlled laboratory experiments, the comparison of methods in environmental samples from Lake Mondsee revealed method‐dependent spatial and temporal variation in abundance patterns. These patterns were species‐dependent, with pronounced discrepancies for *U. castalia* and more subtle, gradient‐specific differences for *U. pseudofurcata*. Such discrepancies may reflect fundamental differences in what each method captures biologically. CARD‐FISH, which targets ribosomal RNA, may preferentially detect metabolically active cells. This interpretation is supported by the observed association between CARD‐FISH‐derived abundances and environmental gradients, as ciliate abundances increased with chlorophyll, phycocyanin, and oxygen concentrations indicating availability of particularly algal food resources. In contrast, dPCR quantifies gene copies and may therefore detect cells regardless of their metabolic state. Differences between the two approaches may thus reflect how each method responds to environmental gradients and captures different aspects of protistan population dynamics.

However, the elevated abundances of *U. castalia* in the upper water layers were not unexpected, since this species is known to commonly inhabit the epilimnion of Lake Mondsee from summer to end of autumn (Frantal et al. 2022; Qu et al. 2020). Similarly, co‐occurrence network analyses identified *Urotricha* as a key node in epilimnetic communities despite the absence of strong associations with known prey, suggesting that these populations may persist in this habitat even when trophic interactions are weak (Qu et al. 2020). The observed discrepancies may therefore likely arise from methodological constraints inherent to each approach. Detection by CARD‐FISH depends on the preservation of intact, ribosome‐containing cells and can therefore be affected by fixation or storage conditions, particularly for fragile ciliates (Piwosz et al. 2021). In addition, fixation‐induced cross‐linking between nucleic acids may have reduced probe accessibility to ribosomal RNA and could have contributed to the underestimation of target cells in the analyses. This may explain observations on our filters where for several *Urotricha* cells only extrusomes emitted a signal (Figure 5C). Furthermore, CARD‐FISH signal intensity in environmental samples was considerably weaker than in laboratory experiments, likely reflecting lower ribosome content in natural populations. If ciliates exhibit reduced metabolic activity, a corresponding decrease in ribosome numbers may limit probe hybridization and increase the likelihood of false negatives. In addition, repeated incubation and washing steps during the hybridization procedure may cause physical loss of weakly attached cells from filters, particularly in complex environmental matrices. Because the *U. castalia* probes were designed using 18S rRNA gene sequences of isolates originating from Lake Mondsee (Dirren‐Pitsch et al. 2022), probe mismatch with local populations is unlikely, suggesting that reduced probe accessibility together with cell loss during processing were the decisive factors. In addition, we observed occasional false‐positive signals, likely caused by interactions between tyramides and endogenous peroxidases, which can mimic true probe signals and complicate the identification of target cells, particularly for very small species, and has been discussed previously (Piwosz et al. 2021). Despite being designed to overcome weak signals and background fluorescence as in conventional FISH, our analyses suggest that CARD‐FISH does not completely eliminate such issues.

Differences between methods may also partly arise from characteristics of molecular approaches. Although we established a species‐specific correction factor for gene copies per cell from different growth phases, other environmental factors are known to be drivers of intraspecific rRNA gene copy number variation (Lavrinienko et al. 2021). For example, laboratory studies of rRNA gene copy number variation of different ciliate taxa suggest that temperature and nutrition availability might represent environmental cues which can elicit rapid changes (Fu and Gong 2017; Sylvester et al. 2009; Zhu et al. 2005). Because our laboratory experiments were conducted under constant temperature and simplified community conditions, gene copy numbers in natural populations may deviate from those measured in culture. Additionally, part of the dPCR signals may have originated from deceased organisms as DNA released from lysed cells or present as extracellular DNA can persist in the environment and may therefore contribute to elevated dPCR estimates (Yu et al. 2025). Another possibility is that DNA can originate from dormant stages such as cysts. However, since for these ciliate species no dormant stages are known to date (Frantal et al. 2022), and none have been detected on any of the CARD‐FISH filters, we do not believe that gene copy numbers are attributable to them. However, although variation in rDNA copy number may contribute to some uncertainty in cell‐number estimates, we consider it unlikely that this factor alone explains the substantial differences observed for *U. castalia*. Instead, reduced detection efficiency and potential cell loss during CARD‐FISH processing likely represent the dominant causes of discrepancy for *U. castalia* between methods in environmental samples. Additional support for this interpretation comes from the higher residual variability observed for CARD‐FISH in environmental samples, which mirrors the higher technical variation detected during laboratory validation. Together, these results indicate that methodological variability contributes to increased scatter and may lead to underestimation of CARD‐FISH–derived abundances.

For *U. pseudofurcata*, we observed a more congruent pattern between methods, with consistently low abundances across depths and throughout the sampling period. While these findings are consistent with a recent study reporting a preference of this species for winter–spring months (Frantal et al. 2022), the better congruency and higher estimates for CARD‐FISH were initially surprising, given that rRNA gene copy numbers in *U. pseudofurcata* also showed intraspecific variations. However, these were less pronounced than those observed for *U. castalia*, suggesting that abundance estimates for *U. pseudofurcata* may be less sensitive to environmentally driven variability. Other methodological factors may have further contributed to these findings. The smaller cells of *U. pseudofurcata* could have been retained more efficiently on CARD‐FISH filters, potentially reducing cell loss during processing. We also acknowledge the possibility that the probe designed for *U. pseudofurcata* may hybridize with the closely related species *U. furcata* , as both share highly similar 18S rRNA gene sequences in the region targeted by the probe (Frantal et al. 2022) and may therefore result in elevated CARD‐FISH–derived abundance estimates. Although we consider this scenario unlikely, it cannot be entirely excluded because the original probe description does not explicitly address potential cross‐reactivity with this species. As *U. furcata* cultures could not be maintained, this could not be tested directly in our study, and additional specificity tests would therefore be required.

### 4.4 Toward a Joint Quantification Framework for Understanding Protist Abundance in Ecology

This study provides a methodological framework for estimating absolute abundances of protists with highly variable rRNA gene copy numbers, such as ciliates. By translating gene copies into meaningful abundance estimates, together with its improved sensitivity, dPCR has the potential to enable stronger ecological inference by allowing us to distinguish true growth or decline of specific taxa within populations in a time‐dependent manner. While taxon‐specific correction factors might need to be evaluated for the organisms targeted in each study, the approach established here lays the foundation for future quantitative applications across diverse protistan groups.

As with any methodology, dPCR also has certain limitations. While our study highlights the potential of dPCR for quantitative applications in protistan ecology, further work is needed to better understand how environmental factors, physiological states, and potential life stages (e.g., cysts) influence intraspecific rRNA gene copy number variation. Many protists undergo different life stages which may differ substantially in their rRNA gene copy numbers (Zou et al. 2021). Consequently, it remains unclear to what extent these different life stages may obscure abundance estimates. In aquatic systems, however, dormant stages are often considered relatively rare within the water column, as they typically sink to the sediments until environmental conditions become more favourable again. It has also been repeatedly shown that rRNA gene copy number may vary depending on the stage of the nuclear cycle the cell is currently in, which in turn is often influenced by environmental factors such as temperature and light intensity (Dapena et al. 2015; Lavrinienko et al. 2021). Future studies could therefore use dPCR to investigate environmentally driven variation in rRNA gene copy numbers and help to better resolve the mechanisms underlying this variability. In addition, CARD‐FISH is itself an emerging method that can be subjected to various biases, as described in detail above, and therefore cannot yet be considered a true ‘gold standard’ for protist quantification. Consequently, interpretations regarding the accuracy of digital PCR in field samples should be made with caution, and future studies may need to further evaluate how dPCR‐based abundance estimates relate to well established quantification methods. Finally, although broader group‐specific correction factors may be applicable for some protistan groups (Girard et al. 2024; Martin et al. 2022), the protocol established here will likely be most useful for the targeted monitoring of key taxa, rather than whole community assessment.

Improving our understanding of mechanisms driving rRNA gene copy number variation in natural protist communities may open new opportunities for integrating molecular and microscopy‐based approaches. Although numerous studies have reported that quantification estimates derived from morphological and molecular approaches rarely coincide (Pröschold et al. 2021; Stoeck et al. 2014), considering both methodologies remains important because they may provide complementary insights into a species realized niche (Frantal et al. 2022; Sonntag et al. 2011). If protists modulate rRNA gene copy numbers in response to environmental cues, combining dPCR with CARD‐FISH could provide complementary information on both genomic and physiological states of populations. For instance, stable cell numbers detected by CARD‐FISH together with increasing gene copy numbers measured by dPCR, potentially accompanied by changes in CARD‐FISH signal intensity reflecting ribosome content, might indicate rapid ecological responses to environmental change that would remain undetected when using either method alone. Regarding method optimization particularly from molecular perspectives, one key aspect is the identification of the involved taxa down to the species level. Knowing a ciliate's name opens up a huge library of ecological background information already collected in the past decades (e.g., Foissner et al. 1999). Moreover, morphological details reveal how a ciliate is adapted to its environment, for example, if it is a crawling species living in biofilm or if it is a floating or jumping species which prefers living in a planktonic habitat. From the characteristics of their cytostome and oral ciliature, additional conclusions on their preferred feeding mode can be recognized which is important when ecological aspects are considered. Finally, dPCR may offer advantages detecting organisms that occur at very low abundances. Many microbial eukaryotes belong to the so‐called rare biosphere, where taxa may occur at concentrations as low as one cell per 100 L of lake water (Logares et al. 2015). At such low densities, detection using microscopy‐based approaches becomes increasingly unlikely, whereas dPCR may still detect target DNA molecules. Applying dPCR could help resolve open questions surrounding rare taxa, which are increasingly recognized as important contributors to functional diversity and ecosystem resilience (Ramond et al. 2025).

### 4.5 Concluding Remarks

Rather than viewing these methodological differences as a limitation of either approach, both may highlight complementary strengths. Digital PCR offers a comparatively simple and scalable workflow that allows high‐throughput processing of many samples, making it well suited for monitoring rapid temporal or spatial dynamics and for detecting low‐abundance or rare taxa due to its high sensitivity. In contrast, CARD‐FISH provides direct visualization and enumeration of intact, ribosome‐containing cells and therefore offers stronger evidence of biologically active populations. Additionally, the possibility of simultaneous hybridization with multiple probes allows investigation of interactions among microbial taxa within the food web. Consequently, the key question is not which method is superior for abundance estimation, but rather under which circumstances each approach is most informative. Used together, dPCR and CARD‐FISH provide complementary perspectives that can improve our understanding of protist population dynamics in natural ecosystems.

## Author Contributions

M.G. conceptualized and designed the study, performed all experiments and data analyses, created the visualizations, and drafted the original manuscript. U.K. maintained laboratory cultures. B.S. provided resources, enabled field sampling, and provided feedback on the final manuscript. T.S. contributed to study conception and design, secured funding, and provided feedback on the final manuscript. All authors approved the final version of the manuscript.

## Funding

T.S. received funding from the Deutsche Forschungsgemeinschaft (grant STO 414/13‐3).

## Conflicts of Interest

The authors declare no conflicts of interest.

## Supporting information


**S1.1:** Overview of gradient end‐point PCR conditions for the evaluation of primer pairs.
**S1.2:** Sequences of synthetic oligonucleotides.
**S1.3:** dPCR scatterplots to test absence of Internal Positive Control in samples.
**S1.4:** List of chemicals and buffers that were used in CARD‐FISH.
**S1.5:** Distance‐Matrix for specificity check of UC833‐1210 primer.
**S1.6:** Distance‐Matrix for specificity check of UC607‐747 primer.
**S1.7:** Distance‐Matrix for specificity check of UP1433‐1789 primer.
**S1.8:** Distance‐Matrix for specificity check of UP1596‐1789 primer.
**S1.9:** Gradient dPCR scatterplots for annealing temperature optimization of primers UC833‐1210 and UC607‐747.
**S1.10:** Gradient dPCR scatterplots for annealing temperature optimization of primers UP1433‐1789 and UP1596‐1789.
**S1.11:** Limit of detection for UC833‐1210 primer.
**S1.12:** Limit of detection for UC607‐747 primer.
**S1.13:** Limit of detection for UP1433‐1789 primer.
**S1.14:** Limit of detection for UP1596‐1789 primer.
**S1.15:** Boxplot of single‐cell gene copy numbers per growth phase for both species.
**S1.16:** Measured gene copy numbers of Internal Positive Control.
**S1.17:** Microphotographs of CARD‐FISH overlays for probe specificity.
**S1.18:** Fixed effects from linear mixed‐effects models testing technical precision.
**S1.19:** Coefficient of variation between independent workflow replicates.
**S1.20:** Fixed effects from linear mixed‐effects models testing workflow variability.
**S1.21:** Fixed effects from linear mixed‐effects models testing measurement bias.
**S1.22:** Summary of linear mixed‐effects models testing the effects of method, depth, sampling date, and environmental gradients on log‐transformed abundance data.

## Data Availability

The data that support the findings of this study are openly available in From gene copies to cell numbers: advancing quantitative app at https://doi.org/10.5281/zenodo.19147375.
