# Supplementary material for: From Gene Copies to Cell Numbers: Advancing Quantitative Approaches in Protistan Ecology Using Digital PCR
Source: Mol Ecol Resour. 2026 Jul 2;26(5):e70177. doi: 10.1111/1755-0998.70177 (PMC13324402; doi:10.1111/1755-0998.70177)
Supplement: Supplementary file 1 — S1.1: Overview of gradient end‐point PCR conditions for the evaluation of primer pairs. S1.2: Sequences of synthetic oligonucleotides. S1.3: dPCR scatterplots to test absence of Internal Positive Control in samples. S1.4: List of chemicals and buffers that were used in CARD‐FISH. S1.5: Distance‐Matrix for specificity check of UC833‐1210 primer. S1.6: Distance‐Matrix for specificity check of UC607‐747 primer. S1.7: Distance‐Matrix for specificity check of UP1433‐1789 primer. S1.8: Distance‐Matrix for specificity check of UP1596‐1789 primer. S1.9: Gradient dPCR scatterplots for annealing temperature optimization of primers UC833‐1210 and UC607‐747. S1.10: Gradient dPCR scatterplots for annealing temperature optimization of primers UP1433‐1789 and UP1596‐1789. S1.11: Limit of detection for UC833‐1210 primer. S1.12: Limit of detection for UC607‐747 primer. S1.13: Limit of detection for UP1433‐1789 primer. S1.14: Limit of detection for UP1596‐1789 primer. S1.15: Boxplot of single‐cell gene copy numbers per growth phase for both species. S1.16: Measured gene copy numbers of Internal Positive Control. S1.17: Microphotographs of CARD‐FISH overlays for probe specificity. S1.18: Fixed effects from linear mixed‐effects models testing technical precision. S1.19: Coefficient of variation between independent workflow replicates. S1.20: Fixed effects from linear mixed‐effects models testing workflow variability. S1.21: Fixed effects from linear mixed‐effects models testing measurement bias. S1.22: Summary of linear mixed‐effects models testing the effects of method, depth, sampling date, and environmental gradients on log‐transformed abundance data. [file MEN-26-e70177-s001.pdf]

MOLECULAR ECOLOGY RESOURCES

Supplemental Information for:

From gene copies to cell numbers: advancing quantitative approaches in protistan ecology using digital PCR

Megan Gross[1*], Ulrike Koll[2], Bettina Sonntag[2], Thorsten Stoeck[1]

[1]Department of Ecology, Rheinland-Pfälzische Technische Universität Kaiserslautern-Landau, Kaiserslautern, Germany

[2]Research Department for Limnology, Mondsee, University of Innsbruck, Mondsee, Austria

*corresponding author

Table of Contents:

**S1.1** Gradient end-point PCR	Page 3
**S1.2** Sequences of synthetic oligonucleotides	Page 4
**S1.3** dPCR test for absence of Internal Positive Control (IPC)	Page 5
**S1.4** List of chemicals and buffers for CARD-FISH	Page 6-7
**S1.5** Distance Matrix for UC833-1210 primer	Page 8
**S1.6** Distance Matrix for UC607-747 primer	Page 9-10
**S1.7** Distance Matrix for UP1433-1789 primer	Page 11

**S1.8** Distance Matrix for UP1596-1789 primer	Page 12
**S1.9** Gradient dPCR for UC833-1210 and UC607-747	Page 13
**S1.10** Gradient dPCR for UP1433-1789 and UP1596-1789	Page 14
**S1.11** Limit of detection UC833-1210	Page 15
**S1.12** Limit of detection UC607-747	Page 16
**S1.13** Limit of detection UP1433-1789	Page 17
**S1.14** Limit of detection UP1596-1789	Page 18
**S1.15** Single-cell gene copy number per growth phase	Page 19
**S1.16** IPC gene copy numbers	Page 20
**S1.17** CARD-FISH probe specificity	Page 21
**S1.18** Linear mixed-effects models for technical precision	Page 22
**S1.19** Coefficient of variation showing workflow variability for both methods	Page 23
**S1.20** Linear mixed-effects models for variability of workflow	Page 24
**S1.21** Linear mixed-effects models for relative error	Page 25
**S1.22** Linear mixed-effects models for environmental samples	Page 26

S1.1:

Gradient-end point PCR was performed on an Eppendorf Master-cycler gradient (Eppendorf, Wesseling-Berzdorf, Germany) with the following conditions: initial denaturation at 95°C for 5 min, followed by 30 cycles of denaturation at 95°C for 15s, annealing at a temperature gradient of 53-62°C with 1°C increments for 15s, and elongation at 68°C for 15s, and a final elongation at 68°C for 5min. The reaction consisted of 10µL 10x ThermoPol reaction buffer (1X f.c.), 1µL dNTPs (200µm f.c.), 1µL of each forward- and reverse-primer (0.2 µm each f.c.), 0.5µL Taq-Polymerase (1.25 units), 2µL DNA and nuclease-free water (reagents from New England Biolabs GmbH, Frankfurt am Main, Germany).

S1.2: Sequences of synthetic oligonucleotides

UC833-1210 (398 base pairs):

5' - GGC TCT CGT ATT GCA AAG CTA GAG GTG AAA TTC TTG GAT TTT TGC AAG ACG AAC TAA TGC GAA AGC ATT TGC CAA GGA TGT TTT CAT TAA TCA AGA ACG AAA GTT AGG GGA TCA AAG ACG ATC AGA TAC CGT CCT AGT CTT AAC TAT AAA CTA TAC CGA CTA GGG ATC AGC CAG GTT TAT CTT AGC TTG GTT GGC ACC TTA TGA GAA ATC AAA GTC TTT GGG TTC TGG GGG GAG TAT GGT CGC AAG GCT GAA ACT TAA AGG AAT TGA CGG AAG GGC ACC ACC AGG CGT GGA GCC TGC GGC TTA ATT TGA CTC AAC ACG GGG AAA CTT ACC AGG TCA AAA CAT GGT TGG GAT TGA CAG ATT GAG AGC TCT TTC TTG ATT CTA TGG GTG GTG GTG CAT GG – 3'

UC607-747 (141 base pairs):

5' - TGG TGT CGA TCT TAG CTG GCT TCG GTC GTT AAT ATC GAT ATC ATC CGT TAG AGA ACT ATA GCC GTC CTT AAC TGG TCA CTA TGG AGA TCT AAC ATT TTA CTT TGA AAA AAT TAG AGT GTT CAA GGC AGG CAA TCG TCA TGA – 3'

UP1443–1789 (369 baise pairs):

5' - GTT TCA CCC TGG CCT GGA AAG GTT TCG GGT AAT CTT TAT AGT ACG TAT CGT GCT AGG GAT CGA TCT TTG GAA TTA TAG ATC TTG AAC GAG GAA TTC CTA GTA AGC GCA TGT CAT CAG CAT GTG CTG ATT ACG TCC CTG CCC TTT GTA CAC ACC GCC CGT CGC TCC TAC CGA TTT CGA GTG ATT CGG TGA ACC TTC TGG ACT ACC TAA GTC CTT GAG TCT TAG GTG GGA AGT TAA GTA AAC CAT ATC ACT TAG AGG AAG GAG AAG TCG TAA CAA GGT ATC CGT AGG TGA ACC TGC GGA TGG ATC ATT AAC ACA AAT CCT ATT TTA CAA ACC TAA TTT ATA TTT TCT TTT GGA GTA TGT GTG CGT AAA GCA – 3'

UP1597–1789 (216 baise pairs):

5' – CCC GTC GCT CCT ACC GAT TTC GAG TGA TTC GGT GAA CCT TCT GGA CTA CCT AAG TCC TTG AGT CTT AGG TGG GAA GTT AAG TAA ACC ATA TCA CTT AGA GGA AGG AGA AGT CGT AAC AAG GTA TCC GTA GGT GAA CCT GCG GAT GGA TCA TTA ACA CAA ATC CTA TTT TAC AAA CCT AAT TTA TAT TTT CTT TTG GAG TAT GTG TGC GTA AAG CAT – 3'

MOLECULAR ECOLOGY
RESOURCES

S1.3: D1 and D2 represent dPCR scatterplots were primer pairs that are specific for the ASV used as internal positive control (referring to the bacterial strain Desulfuromonadaceae) were tested on our lab control (D1) and environmental sample (D2). D3 represents the non-template control. A common threshold was set at 55 RFU.

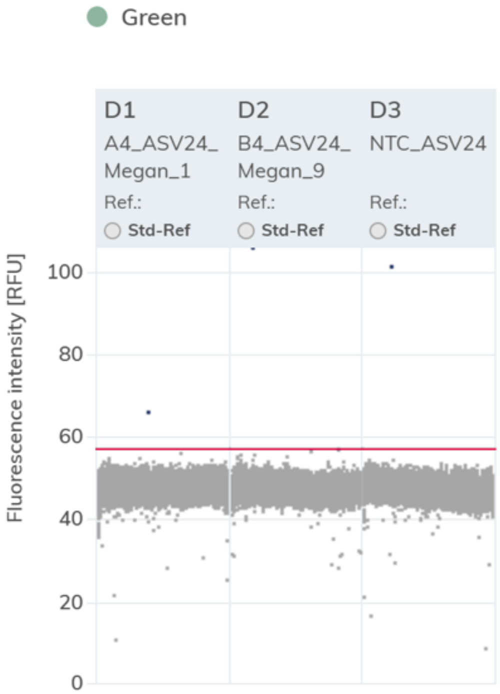

S1.4 List of chemicals for CARD-FISH

Chemical	*Further specification*	*Company*
Formaldehyde	37 % (f.c. 2%)	Carl Roth GmbH + Co. KG, Karlsruhe, Germany
Polycarbonate membrane filters	Isopore™ (pore size 2.0µm; 25mm diameter)<br><br>Isopore™ (pore size 3.0µm, 47mm diameter)	Merck Millipore, Darmstadt, Germany
10x PBS RotiCell		Carl Roth GmbH + Co. KG, Karlsruhe, Germany
Agarose	NEEO	Carl Roth GmbH + Co. KG, Karlsruhe, Germany
HCl	CHEMSOLUTE®	Th. Geyer HmbH &Co. KG,
Oligonucleotide Probes	5'-end tagged with HRP	biomers.net GmbH, Ulm, Germany
Blocking reagent		Roche Diagnostics GmbH, Mannheim, Germany
Maleic acid	HPLC	Merck Sigma Aldrich, Darmstadt, Germany
Tris	1M, pH 7.4	Carl Roth GmbH + Co. KG, Karlsruhe, Germany
Dextran sulfate 500		Carl Roth GmbH + Co. KG, Karlsruhe, Germany
Formamide	Rotipuran ≥ 99.5%	Carl Roth GmbH + Co. KG, Karlsruhe, Germany
SDS (sodium dodecyl sulfate)		Carl Roth GmbH + Co. KG, Karlsruhe, Germany
EDTA	0.5M, pH 8	Thermo Fisher Scientific, Waltham, MA, USA
Triton X-100		Merck Sigma Aldrich, Darmstadt, Germany

H_2O_2		VWR International GmbH, Darmstadt, Germany
Dimethylformamide		Merck Sigma Aldrich, Germany
Triethylamine		Merck Sigma Aldrich, Germany
Tyramine-HCl		Merck Sigma Aldrich, Germany
NHS- Fluorescein	excitation max: 490nm, emission max. 525nm	Thermo Fisher Scientific, Waltham, MA, USA
Glycerol Citifluor AF1		Electron Microscopy Sciences, PA, USA
Vectashield		Biozol Diagnostica, Eching, Germany
DAPI	4', 6'-diamidino-2-phenylindole	Merck Sigma Aldrich, Darmstadt, Germany

S1.5: Estimates of Evolutionary Divergence between Sanger sequence derived from UC833-1210 primers targeting *Urotricha castalia* and 18s sequences of the genus *Urotricha*. *Urotricha* sequences were downloaded from NCBI and are given as accession numbers. Accession numbers corresponding to *Urotricha castalia* are highlighted in green. The number of base substitutions per site from between sequences are shown. Standard error estimate(s) are shown above the diagonal. Analyses were conducted using the Maximum Composite Likelihood model [1]. The analytical procedure encompassed 10 coding nucleotide sequences using 1st, 2nd, 3rd, and non-coding positions. The pairwise deletion option was applied to all ambiguous positions for each sequence pair resulting in a final data set comprising 2.340 positions. Evolutionary analyses were conducted in MEGA12 [2].

	Sequence1	MW077182.1	MW077188.1	MW077190.1	MW077192.1	MW077195.1	ON176660.1	ON176661.1
**Sequence1**		0.01367	0.01914	0.01914	0.00350	0.00350	0.01325	0.01382
**MW077182.1**	0.03347		0.01228	0.01328	0.01395	0.01425	0.00614	0.00900
**MW077188.1**	0.04862	0.04790		0.00686	0.01542	0.01534	0.01228	0.01373
**MW077190.1**	0.04862	0.05192	0.02573		0.01556	0.01544	0.01229	0.01419
**MW077192.1**	0.00338	0.05316	0.05897	0.05965		0.00086	0.01409	0.01572
**MW077195.1**	0.00338	0.05444	0.05830	0.05865	0.00118		0.01434	0.01595
**ON176660.1**	0.03168	0.02313	0.04772	0.04834	0.05406	0.05464		0.00919
**ON176661.1**	0.03354	0.03461	0.05286	0.05444	0.05974	0.06001	0.03567	

S1.6: Estimates of Evolutionary Divergence between Sanger sequences derived from UC607-747 primers targeting *Urotricha castalia* and 18s sequences of the genus *Urotricha*. *Urotricha* sequences were downloaded from NCBI and are given as accession numbers. Accession numbers corresponding to *Urotricha castalia* are highlighted in green. The number of base substitutions per site from between sequences are shown. Standard error estimate(s) are shown above the diagonal. Analyses were conducted using the Maximum Composite Likelihood model [1]. The analytical procedure encompassed 10 coding nucleotide sequences using 1st, 2nd, 3rd, and non-coding positions. The pairwise deletion option was applied to all ambiguous positions for each sequence pair resulting in a final data set comprising 2.340 positions. Evolutionary analyses were conducted in MEGA12 [2].

	**Sequence1**	**Sequence2**	**Sequence3**	**Sequence4**	**Sequence5**	**MW077182.1**	**MW077188.1**	**MW077190.1**	**MW077192.1**	**MW077195.1**	**ON176660.1**	**ON176661.1**
**Sequence1**		0.0103540	0.0000000	0.0000000	0.0000000	0.0730596	0.1706822	0.1547900	0.0069103	0.0000000	0.0715601	0.0689316
**Sequence2**	0.0144275		0.0103540	0.0103540	0.0103540	0.0757957	0.1656715	0.1498895	0.0103540	0.0103540	0.0761816	0.0663672
**Sequence3**	0.0000000	0.0144275		0.0000000	0.0000000	0.0730596	0.1706822	0.1547900	0.0069103	0.0000000	0.0715601	0.0689316
**Sequence4**	0.0000000	0.0144275	0.0000000		0.0000000	0.0730596	0.1706822	0.1547900	0.0069103	0.0000000	0.0715601	0.0689316
**Sequence5**	0.0000000	0.0144275	0.0000000	0.0000000		0.0730596	0.1706822	0.1547900	0.0069103	0.0000000	0.0715601	0.0689316
**MW077182.1**	0.1379714	0.1302388	0.1379714	0.1379714	0.1379714		0.1214433	0.1092409	0.0727616	0.0730596	0.0378651	0.0583669
**MW077188.1**	0.2784672	0.2827446	0.2784672	0.2784672	0.2784672	0.2335854		0.0184890	0.1708104	0.1706822	0.1121329	0.0848709
**MW077190.1**	0.2523278	0.2560669	0.2523278	0.2523278	0.2523278	0.2097831	0.0369838		0.1551371	0.1547900	0.0936468	0.0964895
**MW077192.1**	0.0071211	0.0144275	0.0071211	0.0071211	0.0071211	0.1287478	0.2901860	0.2637328		0.0069103	0.0717665	0.0690311
**MW077195.1**	0.0000000	0.0144275	0.0000000	0.0000000	0.0000000	0.1379714	0.2784672	0.2523278	0.0071211		0.0715601	0.0689316
**ON176660.1**	0.1284452	0.1486454	0.1284452	0.1284452	0.1284452	0.0796529	0.2230753	0.1887949	0.1376431	0.1284452		0.0278453

**ON176661.1**	0.1252992	0.1267044	0.1252992	0.1252992	0.1252992	0.0970256	0.1697056	0.1911679	0.1342389	0.1252992	0.0616632	

S1.7: Estimates of Evolutionary Divergence between Sanger sequences derived from UP1433-1789 primers targeting *Urotricha pseudofurcata* and 18s sequences of the genus *Urotricha*. *Urotricha* sequences were downloaded from NCBI and are given as accession numbers. Accession numbers corresponding to *Urotricha pseudofurcata* are highlighted in green. The number of base substitutions per site from between sequences are shown. Standard error estimate(s) are shown above the diagonal. Analyses were conducted using the Maximum Composite Likelihood model [1]. The analytical procedure encompassed 10 coding nucleotide sequences using 1st, 2nd, 3rd, and non-coding positions. The pairwise deletion option was applied to all ambiguous positions for each sequence pair resulting in a final data set comprising 2.340 positions. Evolutionary analyses were conducted in MEGA12 [2].

	**Sequence1**	**Sequence2**	**Sequence3**	**MW077182.1**	**MW077188.1**	**MW077190.1**	**MW077192.1**	**MW077195.1**	**ON176660.1**	**ON176661.1**
**Sequence1**		0.0026529	0.0036917	0.0240791	0.0140224	0.0026529	0.0341969	0.0338674	0.0271119	0.0260405
**Sequence2**	0.0027177		0.0026398	0.0237368	0.0136713	0.0000000	0.0339026	0.0335930	0.0267586	0.0256516
**Sequence3**	0.0054461	0.0027154		0.0243551	0.0139828	0.0026398	0.0346413	0.0343207	0.0274468	0.0262334
**MW077182.1**	0.1084661	0.1049045	0.1083518		0.0127175	0.0191563	0.0102860	0.0103902	0.0051960	0.0071596
**MW077188.1**	0.0550677	0.0520085	0.0550161	0.0965468		0.0113092	0.0150375	0.0149836	0.0122399	0.0136572
**MW077190.1**	0.0027177	0.0000000	0.0027154	0.1368626	0.0892056		0.0232250	0.0230918	0.0201240	0.0208529
**MW077192.1**	0.1421075	0.1382830	0.1419500	0.0820363	0.1128201	0.1557345		0.0008503	0.0103318	0.0118773
**MW077195.1**	0.1384359	0.1346357	0.1382830	0.0836436	0.1122347	0.1538196	0.0017584		0.0103938	0.0119243
**ON176660.1**	0.1228631	0.1191968	0.1227310	0.0351776	0.0947212	0.1421540	0.0833604	0.0838812		0.0072278
**ON176661.1**	0.1151814	0.1115815	0.1150592	0.0531932	0.1037341	0.1434007	0.0926924	0.0926060	0.0548549	

S1.8: Estimates of Evolutionary Divergence between Sanger sequences derived from UP1596-1789 primers targeting *Urotricha pseudofurcata* and 18s sequences of the genus *Urotricha*. *Urotricha* sequences were downloaded from NCBI and are given as accession numbers. Accession numbers corresponding to *Urotricha pseudofurcata* are highlighted in green. The number of base substitutions per site from between sequences are shown. Standard error estimate(s) are shown above the diagonal. Analyses were conducted using the Maximum Composite Likelihood model [1]. The analytical procedure encompassed 10 coding nucleotide sequences using 1st, 2nd, 3rd, and non-coding positions. The pairwise deletion option was applied to all ambiguous positions for each sequence pair resulting in a final data set comprising 2.340 positions. Evolutionary analyses were conducted in MEGA12 [2].

	Sequence1	Sequence2	Sequence3	MW077182.1	MW077188.1	MW077190.1	MW077192.1	MW077195.1	ON176660.1	ON176661.1
**Sequence1**		0.0000000000	0.0000000000	0.0810581909	0.0525297505	0.0000000000	0.1328792976	0.1318054817	0.0580180101	0.0921435632
**Sequence2**	0.0000000000		0.0000000000	0.0810581909	0.0525297505	0.0000000000	0.1328792976	0.1318054817	0.0580180101	0.0921435632
**Sequence3**	0.0000000000	0.0000000000		0.0810581909	0.0671746486	0.0096096018	0.1328792976	0.1318054817	0.0580180101	0.0921435632
**MW077182.1**	0.1640072801	0.1640072801	0.1640072801		0.0142618133	0.5812439838	0.0156083414	0.0157396505	0.0060699810	0.0096230988
**MW077188.1**	0.1344678571	0.1344678571	0.1566683143	0.0757743766		0.5043013706	0.0165445592	0.0166740155	0.0149931306	0.0167174783
**MW077190.1**	0.0000000000	0.0000000000	0.0185049606	0.7192386781	0.6035596714		0.6063045162	0.6099749144	0.5776931841	0.5729563051
**MW077192.1**	0.1893783517	0.1893783517	0.1893783517	0.0829328145	0.0866449811	0.7705260530		0.0008782502	0.0166992011	0.0188683221
**MW077195.1**	0.1824998730	0.1824998730	0.1824998730	0.0850829967	0.0860639504	0.7716528272	0.0017586275		0.0170505875	0.0194323328
**ON176660.1**	0.1406190017	0.1406190017	0.1406190017	0.0353541230	0.0766905938	0.7172280058	0.0872188693	0.0881694533		0.0102619462
**ON176661.1**	0.1842006383	0.1842006383	0.1842006383	0.0535982492	0.0831387172	0.7058587296	0.0952684069	0.0957577413	0.0552448190	

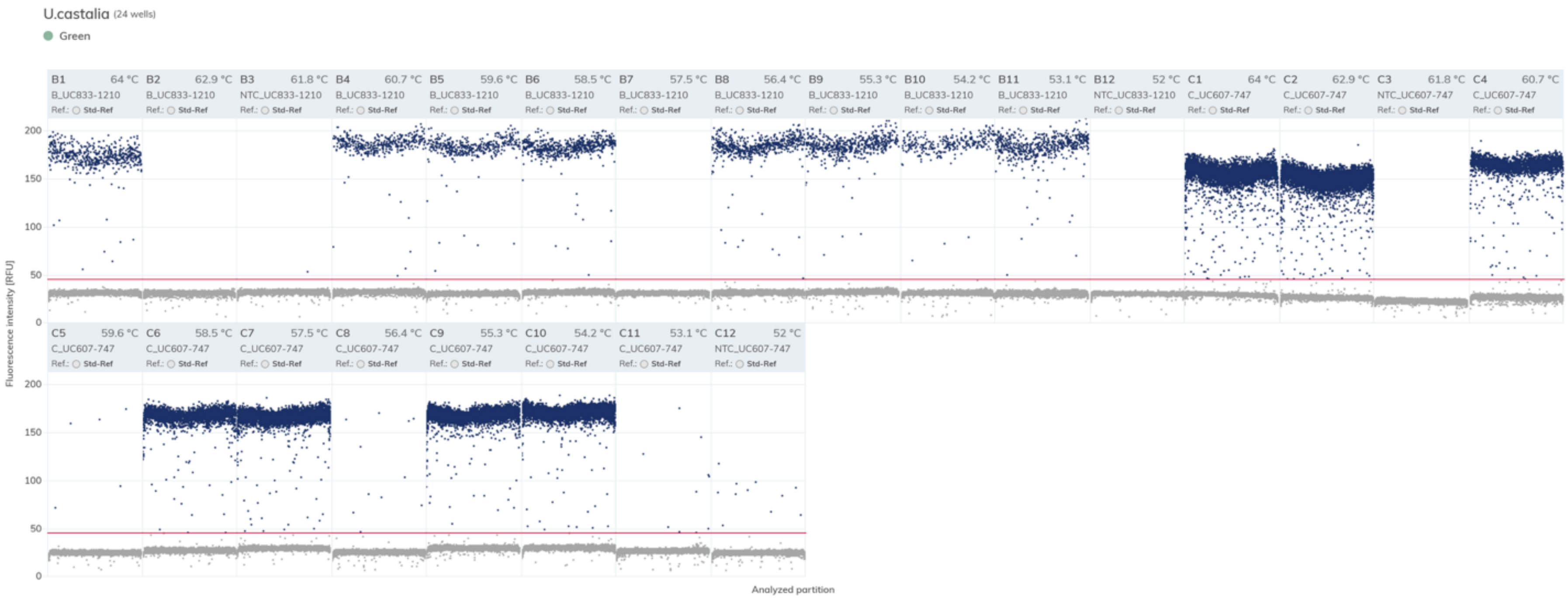

S1.9: Scatterplots of gradient dPCR for *U. castalia* using primers UC833-1210 (B1-B12) and UC607-747 (C1-C12) with a common threshold of 45 RFU.

MOLECULAR ECOLOGY
RESOURCES

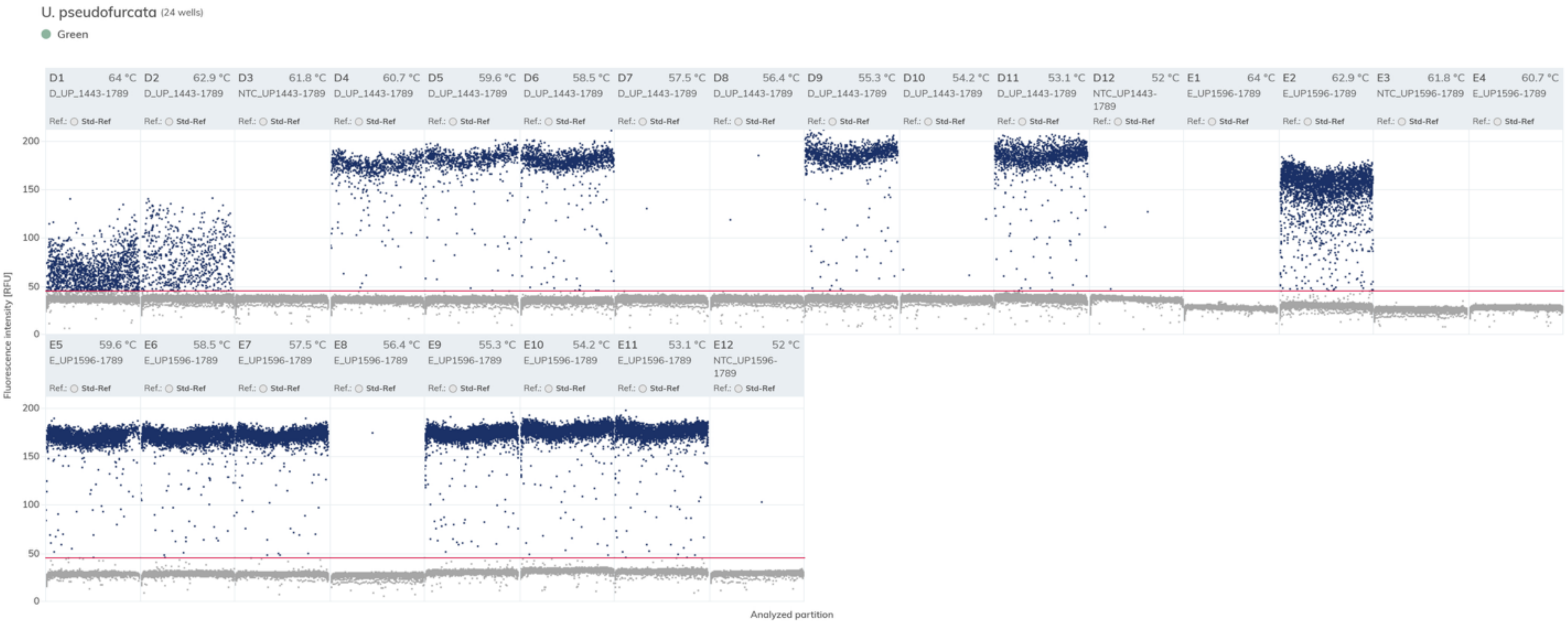

S1.10 Scatterplots of gradient dPCR for *U. pseudofurcata* using primers UP1433-1789 (D1-D12) and UP1596-1789 (E1-E12) with a common threshold of 45 RFU.

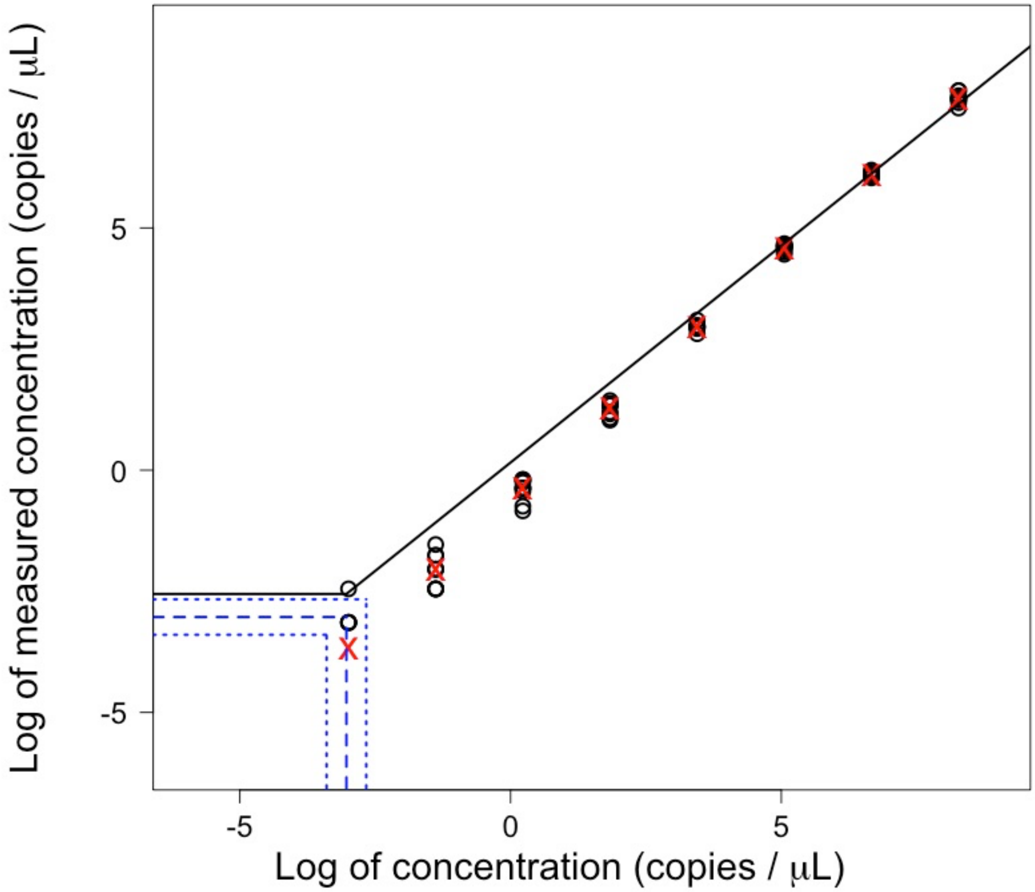

S1.11: Estimated limit of detection (LOD) for UC833-1210, based on a 1:5 serial dilution of synthetic oligonucleotides across eight concentration points with 10 replicates each. Individual replicate measurements are shown as black circles; red Xs denote the mean measured concentration at each dilution level. The LOD is defined as the point of intersection between the linear regression fitted to the quantifiable concentrations (solid black line) and the background response plateau where the instrument fails to distinguish signal from noise (dashed blue line, with upper and lower 95% confidence bounds shown as dotted blue lines). The estimated LOD was 0.0482432 copies/µL. An intersection is not visible in the plot because the regression and plateau curves intersect outside the range of measured concentrations.

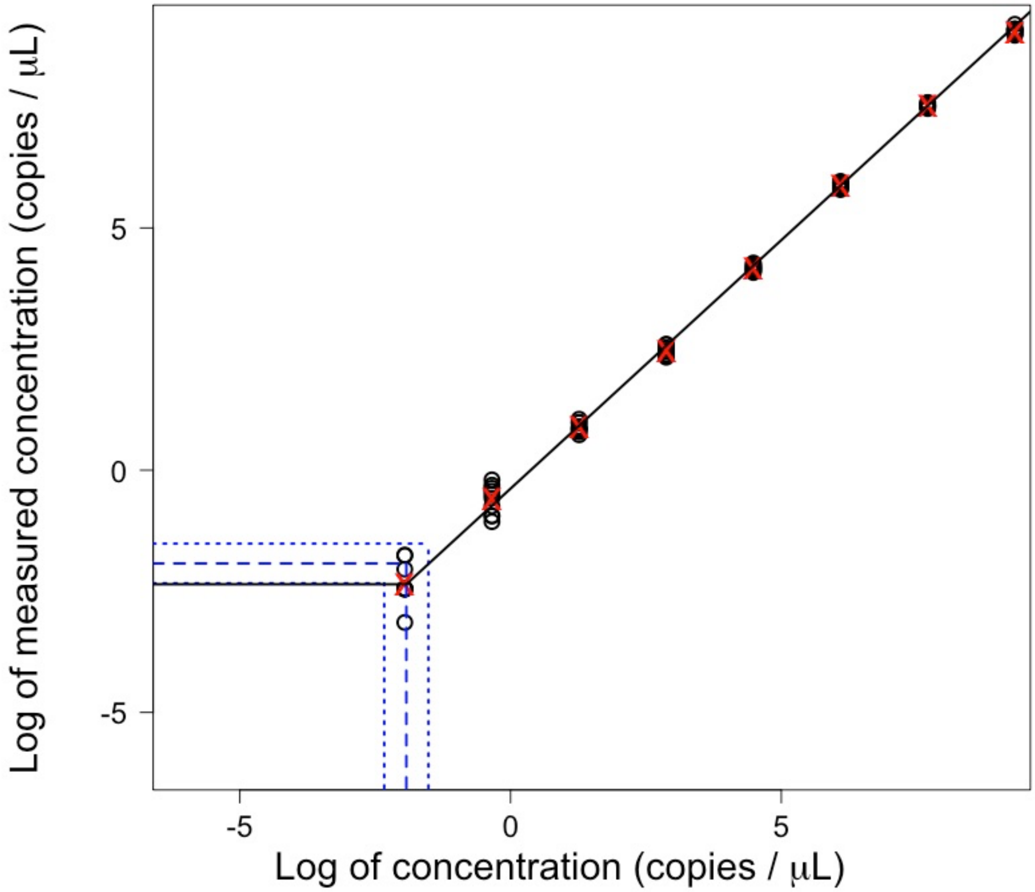

S1.12: Estimated limit of detection (LOD) for UC607-747, based on a 1:5 serial dilution of synthetic oligonucleotides across six concentration points with 10 replicates each. Individual replicate measurements are shown as black circles; red Xs denote the mean measured concentration at each dilution level. The LOD is defined as the point of intersection between the linear regression fitted to the quantifiable concentrations (solid black line) and the background response plateau where the instrument fails to distinguish signal from noise (dashed blue line, with upper and lower 95% confidence bounds shown as dotted blue lines). The estimated LOD was 0.1459074 copies/µL.

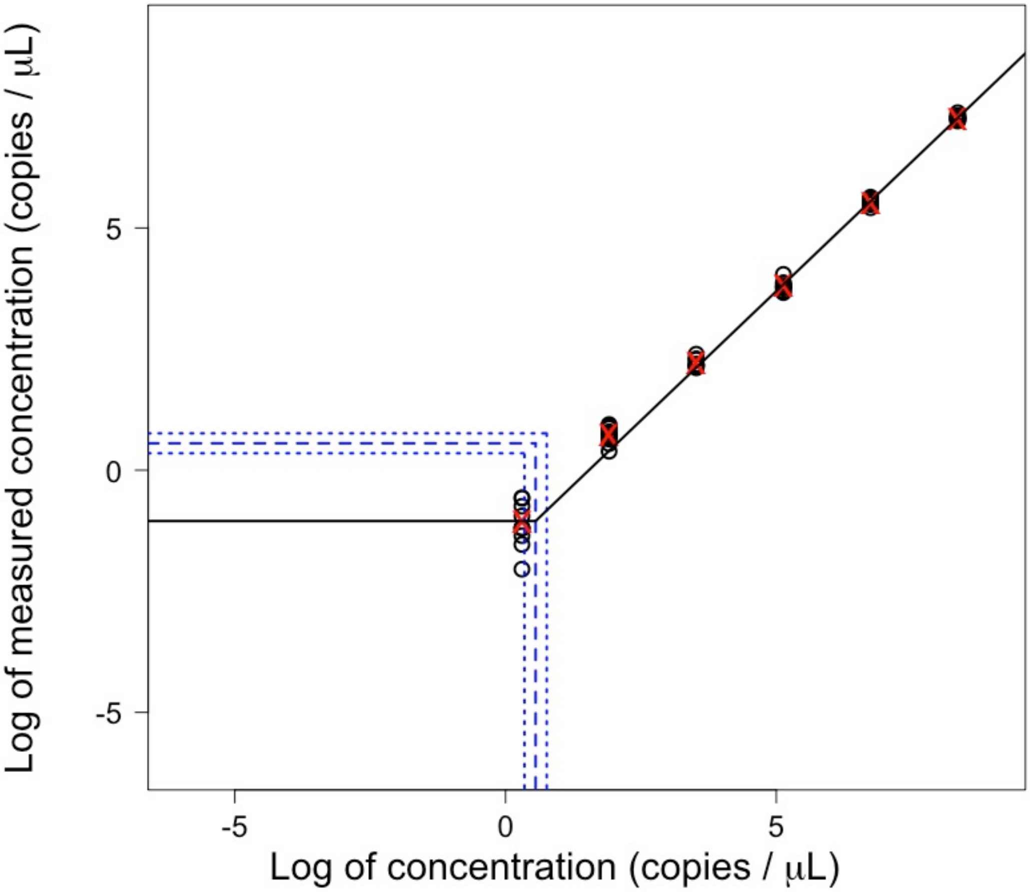

S1.13: Estimated limit of detection (LOD) for UP1433-1789, based on a 1:5 serial dilution of synthetic oligonucleotides across six concentration points with 10 replicates each. Individual replicate measurements are shown as black circles; red Xs denote the mean measured concentration at each dilution level. The LOD is defined as the point of intersection between the linear regression fitted to the quantifiable concentrations (solid black line) and the background response plateau where the instrument fails to distinguish signal from noise (dashed blue line, with upper and lower 95% confidence bounds shown as dotted blue lines). The estimated LOD was 1.741467 copies/µL.

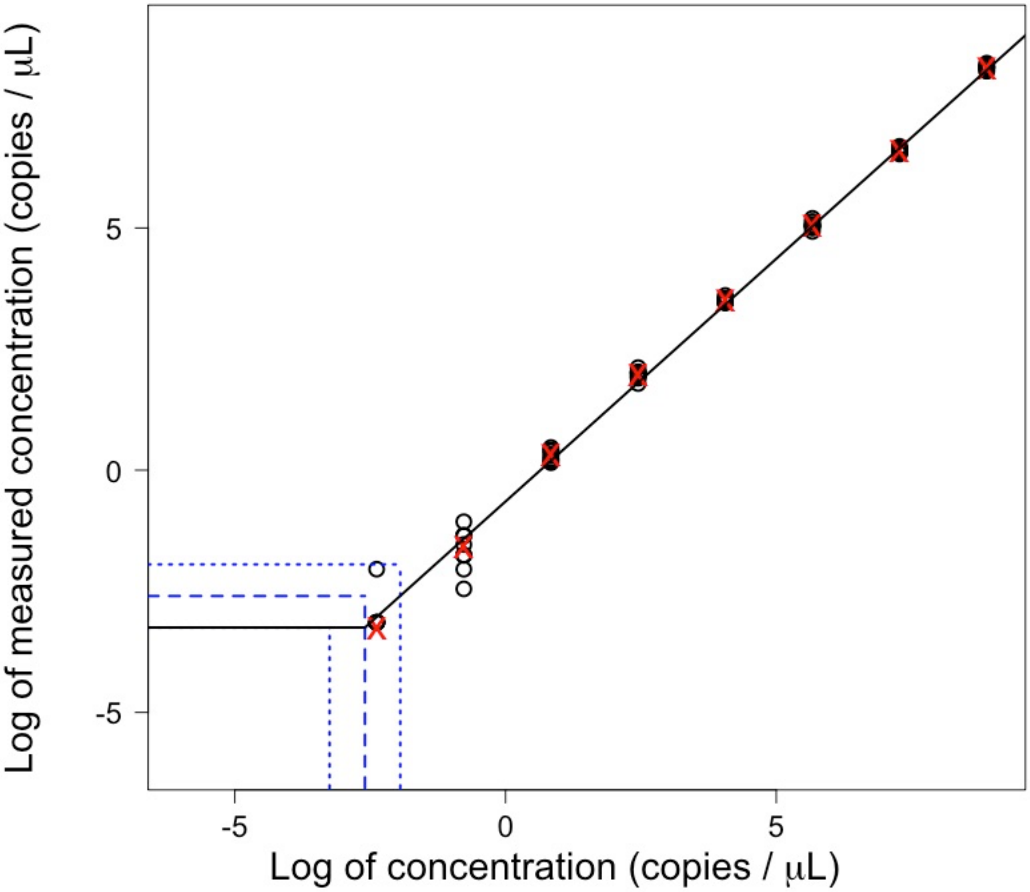

S1.14: Estimated limit of detection (LOD) for UP1596-1789, based on a 1:5 serial dilution of synthetic oligonucleotides across nine concentration points with 10 replicates each. Individual replicate measurements are shown as black circles; red Xs denote the mean measured concentration at each dilution level. The LOD is defined as the point of intersection between the linear regression fitted to the quantifiable concentrations (solid black line) and the background response plateau where the instrument fails to distinguish signal from noise (dashed blue line, with upper and lower 95% confidence bounds shown as dotted blue lines). The estimated LOD was 0.07447753 copies/μL.

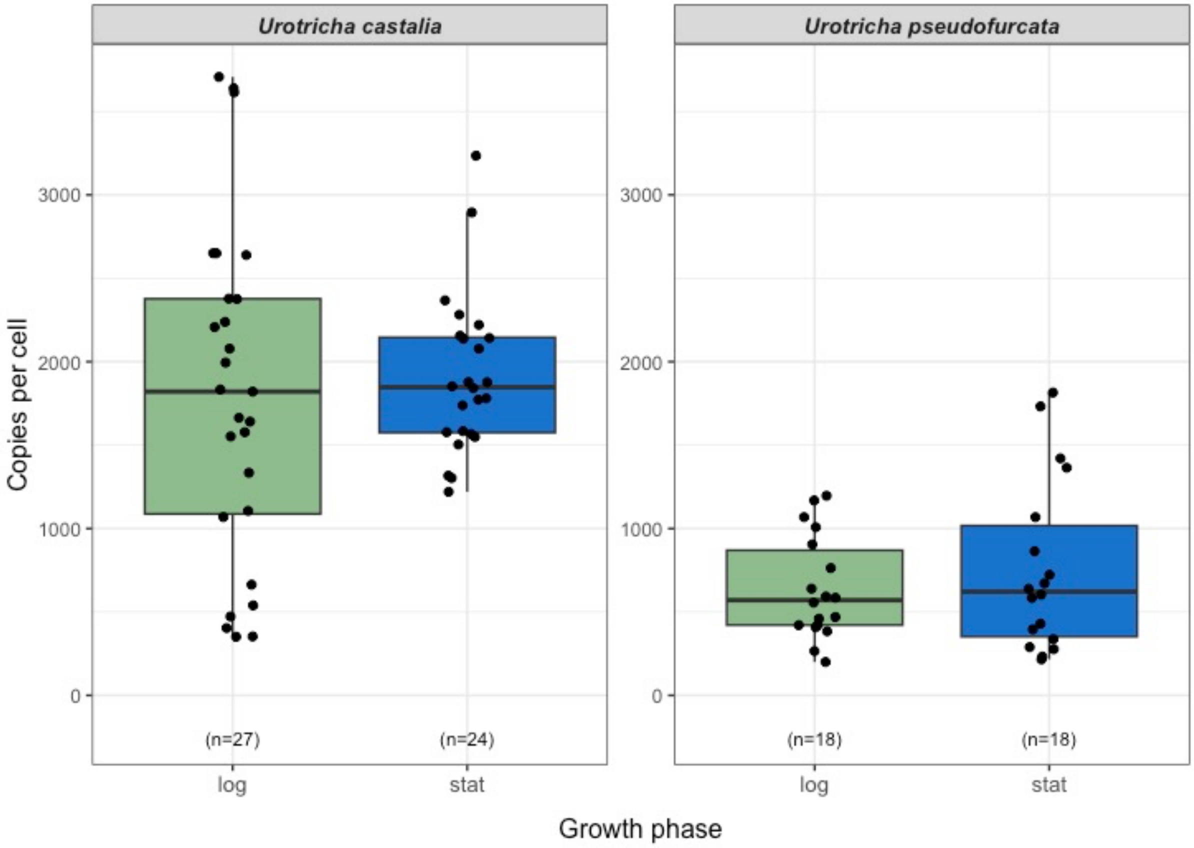

S1.15: Single-cell gene copy numbers per growth phase (logarithmic vs. stationary) for *Urotricha castalia* and *Urotricha pseudofurcata*. Points represent sample-level estimates; boxplots show medians and interquartile ranges.

S1.16: Gene copies of internal positive control. Extraction control refers to gene copy number of synthetic oligonucleotides that were spiked into samples before DNA extraction and non-extraction control refers to gene copies of pure synthetic oligonucleotides. Internal positive control – correction factor was calculated as by dividing the average of non-extraction control by the average of extraction control.

sample	copies(per µl reaction)	copies(per reaction)	copies_IPC	group
**IPC_Mix1.1**	9.61	384.24	38423.73	Extraction control
**IPC_Mix1.1**	8.00	319.88	31988.39	Extraction control
**IPC_Mix1.1**	7.21	288.44	28844.15	Extraction control
**IPC_Mix1.2**	5.84	233.68	23368.29	Extraction control
**IPC_Mix1.2**	5.65	226.13	22612.53	Extraction control
**IPC_Mix1.2**	5.72	228.86	22886.25	Extraction control
**IPC_Mix1.3**	3.86	154.36	15435.87	Extraction control
**IPC_Mix1.3**	3.75	150.16	15016.49	Extraction control
**IPC_Mix1.3**	3.55	141.85	14184.59	Extraction control
**IPC_Mix2.1**	10.26	410.23	41022.72	Extraction control
**IPC_Mix2.1**	10.33	413.23	41322.64	Extraction control
**IPC_Mix2.1**	9.42	376.77	37676.78	Extraction control
**IPC_Mix2.2**	6.55	261.91	26191.29	Extraction control
**IPC_Mix2.2**	9.76	390.58	39058.12	Extraction control
**IPC_Mix2.2**	11.36	454.57	45457.34	Extraction control
**IPC_Mix2.3**	7.42	296.63	29662.88	Extraction control
**IPC_Mix2.3**	9.35	373.96	37395.87	Extraction control
**IPC_Mix2.3**	7.88	315.31	31531.09	Extraction control
**IPC_gBlocks**	880.52	35220.73	352207.32	-non- extraction control
**IPC_gBlocks**	892.18	35687.32	356873.17	-non- extraction control
**IPC_gBlocks**	827.26	33090.49	330904.86	-non- extraction control
**IPC- correction factor**			**11.51**	

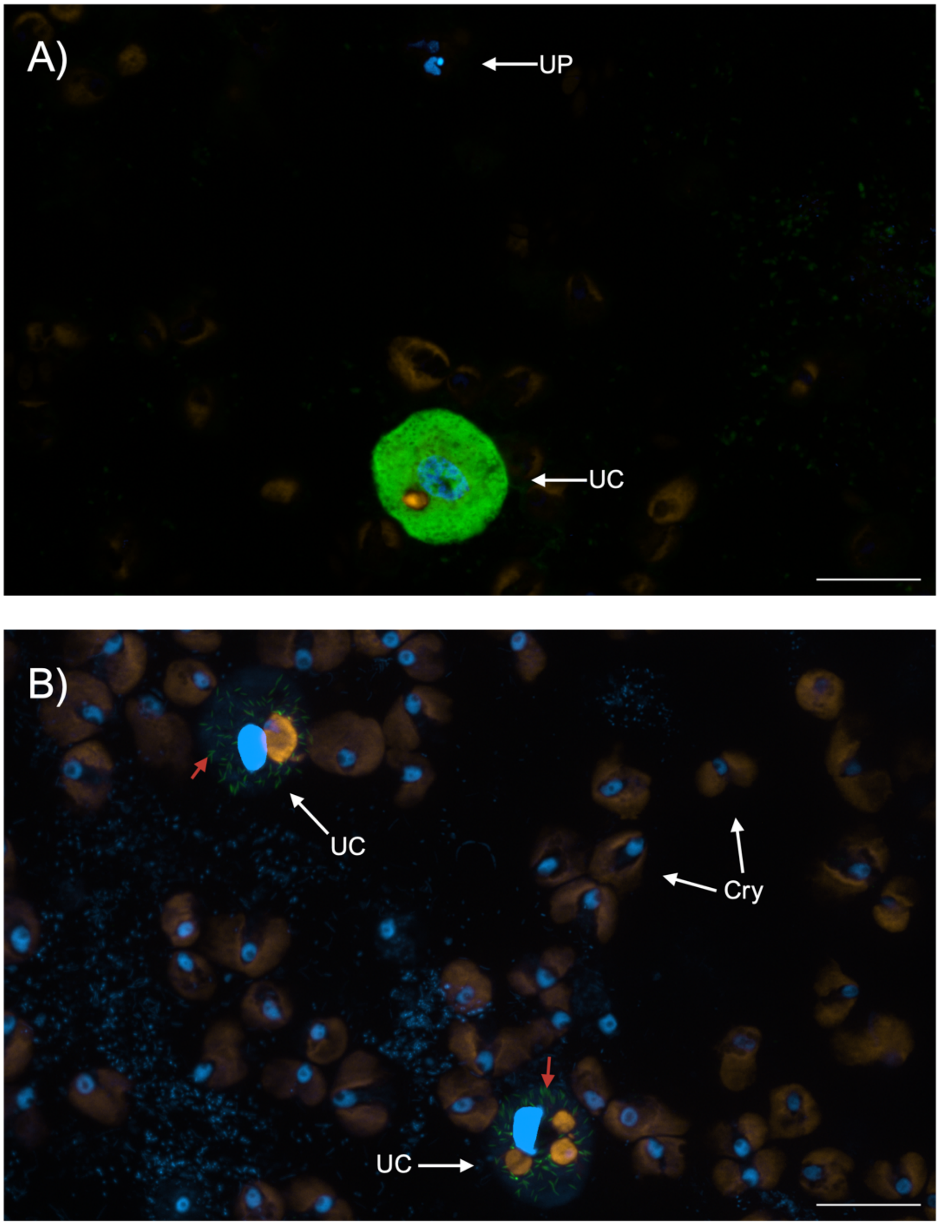

S1.17: Microphotographs of CARD-FISH overlays showing probe-specificity in lab-experiments of mixed Urotricha samples using A) *U. castalia* specific Uro2-1440 probe and B) *U.pseudofurcata* specific Uro5-403 probe. Nuclei are visualized with DAPI (blue). Cryptomonas and food vacuoles emit an autofluorescent signal (red). White arrows point to different organisms. Red arrows in B) point to extrusomes that emit unspecific fluorescent signal. UC= *U. castalia*, UP= *U. pseudofurcata*, Cry = *Cryptomonas* SAG 26.80.

S1.18: Fixed effects from linear mixed-effects models testing technical precision (coefficient of variation [CV] among technical replicates) as a function of method (CARD-FISH vs. dPCR), community richness, log_{10} expected abundance, and their interaction for two ciliate targets (*Urotricha castalia* and *Urotricha pseudofurcata*). Models included random intercepts for sample identity. Significant effects ($p < 0.05$) are indicated in bold.

target	***term***	***Estimate (SE)***	***df***	***statistic***	***p-value***
U. castalia	(Intercept)	0.470 (0.260)	40	1.810	0.078
	Log (expected)	-0.300 (0.085)	40	-3.532	**0.001**
	Method (dPCR vs. CARD-FISH)	-1.097 (0.368)	40	-2.985	**0.005**
	Method x Log (expected)	0.010 (0.120)	40	0.082	0.935
	Richness	-0.060 (0.044)	40	-1.374	0.177
	Richness x Method	0.014 (0.062)	40	0.230	0.819
U. pseudofurcata	(Intercept)	0.668 (0.321)	34	2.082	**0.045**
	Log (expected)	-0.407 (0.108)	34	-3.766	**<0.001**
	Method (dPCR vs. CARD-FISH)	-1.119 (0.454)	34	-2.465	**0.019**
	Method x Log (expected)	0.108 (0.153)	34	0.709	0.483
	Richness	-0.042 (0.055)	34	-0.773	0.445
	Richness x Method	0.059 (0.077)	34	0.766	0.449

S1.19: Coefficient of variation (CV) between independent workflow replicates for both methods and targets. Sd = standard deviation, IQR = interquartile range.

Target	*method*	*N*	*mean CV (%)*	*median CV (%)*	*sd CV (%)*	*IQR*
U. castalia	CARD-FISH	7	28.24	24.05	15.39	21.98
U. castalia	dPCR	7	31.77	30.1	19.22	10.34
U. pseudofurcata	CARD-FISH	7	31.21	23.49	24.84	29.82
U. pseudofurcata	dPCR	7	35.6	27.21	24.94	26.14

S1.20: Fixed effects from linear mixed-effects models testing variability of workflow as a function of method (CARD-FISH vs. dPCR), community richness, $\log_{10}$ expected abundance, and their interaction for two ciliate targets (*Urotricha castalia* and *Urotricha pseudofurcata*). Models included random intercepts for sample identity. Significant effects ($p < 0.05$) are indicated in bold.

target	*term*	*Estimate (SE)*	*df*	*statistic*	*p-value*
U. castalia	(Intercept)	-0.136 (0.614)	8	-0.221	0.830
	Log (expected)	-0.246 (0.152)	8	-1.615	0.145
	Method (dPCR vs. CARD-FISH)	0.173 (0.868)	8	0.199	0.847
	Method x Log (expected)	0.065 (0.215)	8	0.440	0.672
	Richness	-0.126 (0.226)	8	0.559	0.592
	Richness x Method	-0.255 (0.319)	8	-0.800	0.447
U. pseudofurcata	(Intercept)	-1.907 (0.778)	8	-2.453	**0.040**
	Log (expected)	0.138 (0.193)	8	0.718	0.494
	Method (dPCR vs. CARD-FISH)	2.212 (1.1)	8	2.012	0.079
	Method x Log (expected)	-0.238 (0.272)	8	-0.874	0.408
	Richness	0.603 (0.286)	8	2.111	0.068
	Richness x Method	-1.003 (0.404)	8	-2.482	**0.038**

S1.21: Fixed effects from linear mixed-effects models testing measurement bias (mean log_{10}(observed/expected)) as a function of method (CARD-FISH vs. dPCR), community richness, log_{10} expected abundance, and their interaction for two ciliate targets (*Urotricha castalia* and *Urotricha pseudofurcata*). Models included random intercepts for sample identity. Significant effects ($p < 0.05$) are indicated in bold.

target	*term*	*Estimate (SE)*	*df*	*statistic*	*p-value*
U. castalia	(Intercept)	-0.609 (0.264)	16.61	-2.310	**0.034**
	Log (expected)	0.115 (0.087)	15.29	1.321	0.206
	Method (dPCR vs. CARD-FISH)	0.053 (0.320)	34.34	0.164	0.871
	Richness	0.002 (0.042)	29.39	0.041	0.968
	Richness x Method	0.025 (0.054)	34.34	0.458	0.650
U. pseudofurcata	(Intercept)	-0.679 (0.248)	34	-2.743	**0.010**
	Log (expected)	0.182 (0.083)	34	2.184	**0.036**
	Method (dPCR vs. CARD-FISH)	-0.195 (0.350)	34	-0.557	0.581
	Richness	-0.043 (0.042)	34	-1.026	0.312
	Richness x Method	0.040 (0.06)	34	0.676	0.503

S1.22: Summary of linear mixed-effects models testing the effects of method (dPCR vs CARD-FISH), depth, sampling date, and environmental gradients (PCenv2) on log-transformed abundance of *Urotricha castalia* and *Urotricha pseudofurcata*. Estimates (β ± SE) are shown for fixed effects. Test statistics correspond to t-tests for model coefficients and Type III ANOVA F-tests for main and interaction effects. Q1/Q2 models were used to assess method-specific differences in spatial (depth) and temporal (sampling date) abundance patterns, whereas Q3 models tested whether abundance responses to environmental gradients (PCenv2) differed between methods.

Target	***Model***	***Effect***	***Estimate (β ± SE)***	***df***	***Test statistic***	***p-value***
U. castalia	Q1/Q2	Method (dPCR vs CARD-FISH)	1.78 ± 0.24	34	t = 7.46	<0.001
		Depth (hypolimnion)	0.39 ± 0.30	10.6	t = 1.31	0.219
		Method vs. Depth		34	F = 8.68	0.006
		Day (overall)		5.5	F = 0.87	0.56
	Q3	Method (dPCR vs CARD-FISH)	1.28 ± 0.19	34	t = 6.81	<0.001
		PCenv2	~0.00 ± 0.14	6.29	t = -0.003	0.998
		Method vs PCenv2		34	F = 0.14	0.71
		Depth		4	F = 0.18	0.693
		Day (overall)		5.4	F = 0.71	0.65
U. pseudofurcata	Q1/Q2	Method (dPCR vs CARD-FISH)	-0.31 ± 0.21	37	t = -1.50	0.143
		Depth (hypolimnion)	-0.38 ± 0.20	37	t = -1.90	0.066
		Method vs Depth		37	F = 3.89	0.056
		Day (overall)		5.37	F = 2.21	0.074
	Q3	Method (dPCR vs CARD-FISH)	-0.02 ± 0.14	36	t = -0.17	0.863
		PCenv2	0.24 ± 0.08	36	t = 3.08	0.0039
		Method vs PCenv2		36	F = 7.40	0.01
		Depth		36	F = 0.30	0.589
		Day (overall)		5.36	F = 1.21	0.322
